# Leveraging Macromolecular Isomerism for Phase Complexity in Janus Nanograins

**DOI:** 10.1021/acscentsci.2c01405

**Published:** 2023-02-08

**Authors:** Yu Shao, Di Han, Yangdan Tao, Fengfeng Feng, Ge Han, Bo Hou, Hao Liu, Shuguang Yang, Qiang Fu, Wen-Bin Zhang

**Affiliations:** † Beijing National Laboratory for Molecular Sciences, Key Laboratory of Polymer Chemistry & Physics of Ministry of Education, College of Chemistry and Molecular Engineering, Center for Soft Matter Science and Engineering, Peking University, Beijing 100871, China; ‡ College of Polymer Science & Engineering, State Key Laboratory of Polymer Materials Engineering, Sichuan University, Chengdu 610065, China; § Center for Advanced Low-Dimension Materials, State Key Laboratory for Modification of Chemical Fibers and Polymer Materials, Donghua University, Shanghai 201620, China

## Abstract

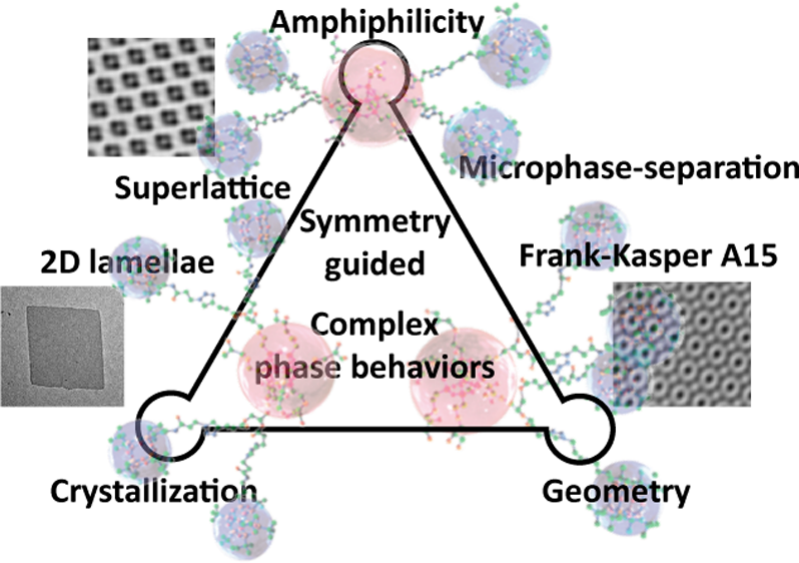

It remains intriguing whether macromolecular isomerism, along with competing molecular interactions, could be leveraged to create unconventional phase structures and generate considerable phase complexity in soft matter. Herein, we report the synthesis, assembly, and phase behaviors of a series of precisely defined regioisomeric Janus nanograins with distinct core symmetry. They are named B_2_DB_2_ where B stands for *iso*-butyl-functionalized polyhedral oligomeric silsesquioxanes (POSS) and D stands for dihydroxyl-functionalized POSS. While BPOSS prefers crystallization with a flat interface, DPOSS prefers to phase-separate from BPOSS. In solution, they form 2D crystals owing to strong BPOSS crystallization. In bulk, the subtle competition between crystallization and phase separation is strongly influenced by the core symmetry, leading to distinct phase structures and transition behaviors. The phase complexity was understood based on their symmetry, molecular packing, and free energy profiles. The results demonstrate that regioisomerism could indeed generate profound phase complexity.

## Abstract 2

In a series of B_2_AB_2_-type Janus nanograins, macromolecular isomerism was combined with competing interactions between crystallization and phase separation to generate considerable phase complexity.

## Introduction

Since its conception by Jacob Berzelius,^1^ molecular isomerism has been recognized as a fundamental phenomenon in chemistry featuring drastic property changes with identical composition. While numerous examples have been documented for small molecules, macromolecular isomerism is a relatively recent subject.^2^ Chain molecules are often polydispersed and rely on collective interactions among multiple regio- or stereocenters to exhibit distinct physical properties.^3,4^ In the study of structure–property relationships in macromolecules, it remains elusive how significant changes could be introduced into macromolecular systems on variation of only a single regio- or stereocenter.^5,6^ The question must be addressed within the framework of precision macromolecules.

Giant molecules are precision macromolecules built on molecular nanoparticles.^7−10^ Unlike synthetic polymers, they are not long-chain molecules, but conformationally rigid molecular nanograins with defined sizes and shapes. Hence, their assembly is sensitive to minute primary structural changes, providing an ideal platform for studying macromolecular isomerism.^11−16^ Previously, a series of bifunctional polyhedral oligomeric silsesquioxane (POSS) regioisomers have been reported,^17,18^ which promotes the investigation of regioconfiguration on the self-assembly of giant molecules, such as Janus star polymers,^18,19^ double-chained giant surfactants,^20,21^ quadruple-chained giant surfactants,^22,23^ Janus amphiphilic particles,^24,25^ giant polymeric chains,^26−28^ molecular patchy clusters,^15^ and particle-like smectic liquid crystals.^29^ Not only does regiochemistry exert profound influences on the equilibrium structures and transition kinetics of their assembly (as in isomeric double-chained surfactants^20,21^ and ABA-type Janus nanoparticles^24,25,29^), but there is also a strong correlation between regiochemistry and the formation of Frank–Kasper phases (as in quadruple-chained giant surfactants,^22^ patchy particle isomers,^15^ and geometric isomers^30^). While the profound role of isomerism in tuning assembly is unambiguously established, challenges remain on whether unconventional phases and considerable phase complexity could be introduced by varying only one regiochemistry in macromolecular isomers.

The emergence of unconventional phases in single-component molecular systems is a recent event that links molecular symmetry/design with molecular interactions/close-packing.^31,32^ To date, such phases include 2D tilings,^33,34^ 3D networks,^35−41^ and spherical-packing such as Frank–Kasper A15 phase and sigma phase,^42−55^ Z phase,^56^ Laves C14 and C15 phases,^57−63^ etc. Recently, a deliberate pairing of giant molecules has led to peculiar self-sorting behavior which opens an avenue for constructing complex soft lattices, such as binary crystal phases^64−67^ and decagonal quasicrystals,^68^ with intriguing dynamic properties.^69^ Despite these successes, molecular isomerism has not yet been well exploited in generating unconventional phases.

Herein, we report the synthesis, assembly, and phase behaviors of a full set of regioisomeric Janus nanograins based on POSS with distinct core symmetry, namely, B_2_DB_2_ where B stands for *iso*-butyl-functionalized polyhedral oligomeric silsesquioxanes (POSS) and D stands for dihydroxyl-functionalized POSS. We envisioned that the interplay between BPOSS crystallization^70^ and BPOSS/DPOSS phase separation^46^ should lead to unconventional phases and complex phase behaviors as a sole consequence of macromolecular isomerism under the constraint of core symmetry and volume asymmetry.

## Results and Discussion

### Molecular Design and Precision Synthesis

**Scheme 1:**
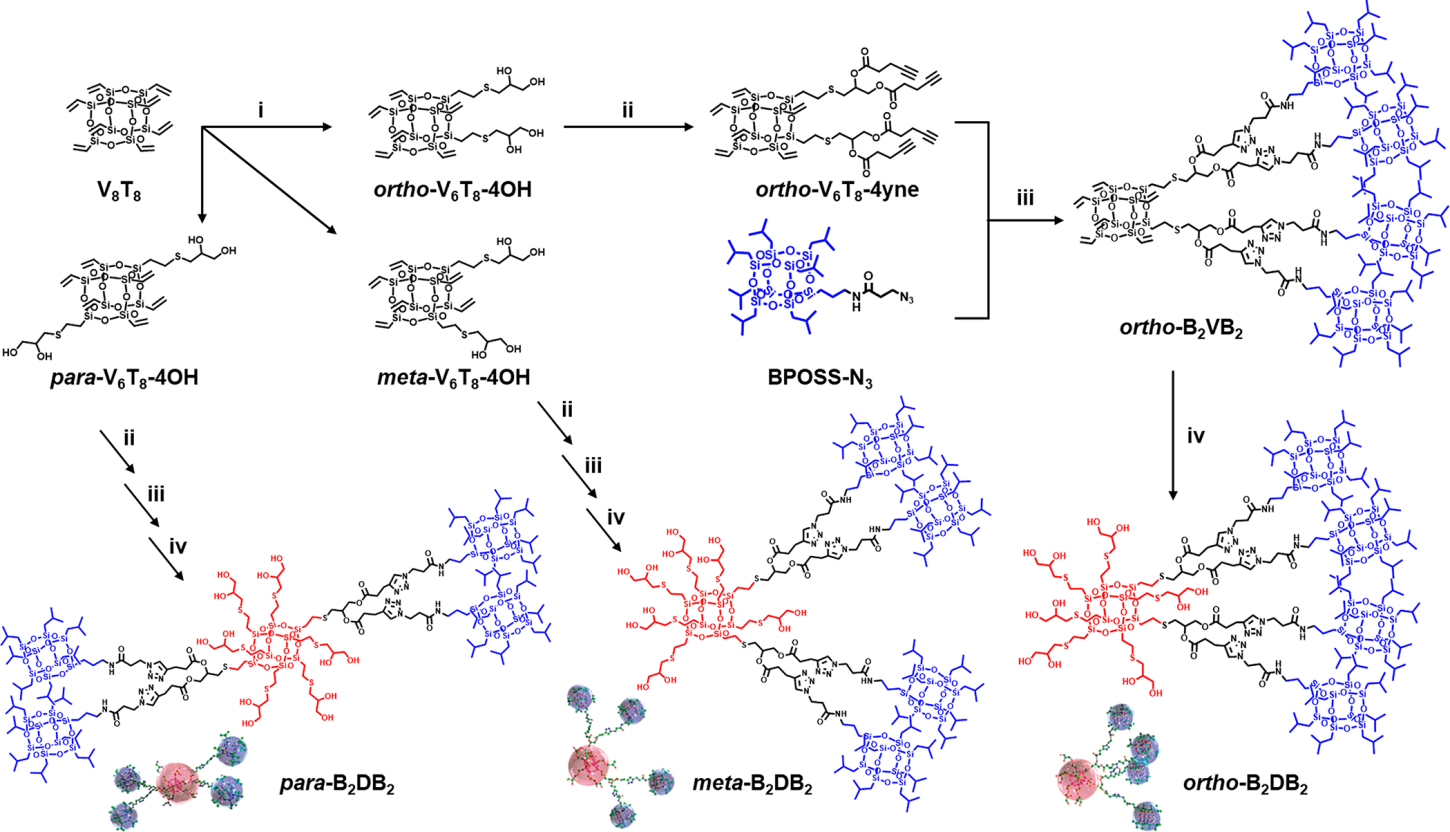
Synthetic Route of B_2_DB_2_ Giant Molecular Isomers Reagent and conditions: (i) 1-Thioglycerol; DMPA; UV (365 nm); 10 min; THF; separation yields for V_6_T_8_-4OH isomers, ∼8% for the *para*-isomer, ∼10% for the *meta*-isomer, and ∼10% for the *ortho*-isomer. (ii) 4-Pentynoic acid; DIPC; DMAP; DCM; 24 h; ∼80%. (iii) BPOSS-N_3_; CuBr; PMDETA; THF; 12 h; ∼70%. (iv) 1-Thioglycerol; Irgacure 2959; UV (365 nm); 10 min; THF; ∼70%.

Previously, we have shown that the reaction between octavinyl POSS and 1-thioglycerol gives a mixture of different adducts from which the three bifunctional POSS isomers can be isolated and fully identified.^22^ These *ortho*-, *meta*-, and *para*-isomers of bifunctional POSS are ideal scaffolds for regioisomer design.^11^ By replacing the soft building blocks in previous examples (e.g., polystyrene chains and noncrystalline POSS) with a crystalline BPOSS, we intended to introduce crystallization as a strong competing force to phase separation. To reach spherical phases, a large volume asymmetry between hydrophilic DPOSS and hydrophobic BPOSS is required, which could be conveniently achieved by controlling a ratio of 1:4 for DPOSS:BPOSS for a final BPOSS volume fraction (*f*_BPOSS_) of ∼0.82 (Scheme 1, Table 1).^46^ Hence, we installed two hydroxyl groups on one vertex of bifunctional POSS for subsequent attachment of one BPOSS per hydroxyl group. A straightforward way to prepare such tetrafunctional POSS is via thiol–ene chemistry using thiolglycerol.^22^ The bifunctional regioisomers (V_6_T_8_-4OH) were converted to V_6_T_8_-4yne and linked with four BPOSS through a copper-catalyzed alkyne–azide cyclization reaction to give B_2_VB_2_ (Scheme 1, see Figures S1 and S2 for ^1^H NMR and ^13^C NMR characterizations).^24^ Then, the vinyl groups were converted to hydroxyl groups to yield the Janus nanograins B_2_DB_2_ by a second thiol–ene reaction (see Figures S3 and S4 for NMR characterizations).

**Table 1:** Physical Property Summary of B_2_DB_2_ Regioisomers

configuration	*f*_BPOSS_^a^	*T*_m_^b^ (°C)	*T*_c_^c^ (°C)	lattice 0^d^	*d*_0_^e^	lattice 1^f^	lattice dimension^g^ (nm)	lattice 2^h^	*a*_2_^i^ (nm)	μ^j^	*A*_0,2_^k^ (nm^*2*^)
*para*	0.82	189	165	iLAM	3.4	hexagonal crystal	*a* = 5.76; *c* = 9.59	Col_h_	5.33	3.2	2.21
*meta*	0.82	186	168	iLAM	3.4	hexagonal crystal	*a* = 5.76; *c* = 9.59	iCol_h_			
*ortho*	0.82	186	165	iLAM	3.0	hexagonal crystal	*a* = 5.58; *c* = 9.38	A15	11.07	21.5	2.16

a Volume fraction of BPOSS.

b Melting temperatures are the extrapolated onset temperatures obtained from DSC.

c Crystallization temperatures are peak values from DSC.

d Frustrated lamellar packing of the samples.

e Layer spacing of the iLAM morphology.

f Lattice 1 represents the structure developed after annealing at the temperature close to *T*_c_.

g Lattice dimensions of lattice 1.

h Lattice 2 represents the phase-separated structure when BPOSS crystal melts.

i Lattice dimension of lattice 2.

j Average number of molecules within a 1 nm thick cross-section of the cylinders in the Col_h_ structure and the calculated average numbers of molecules per supramolecular sphere in the Frank–Kasper A15 structure.

k Average interfacial area per molecule calculated for lattice 2. The detailed calculation can be found in the Supporting Information.

**Figure 1:**
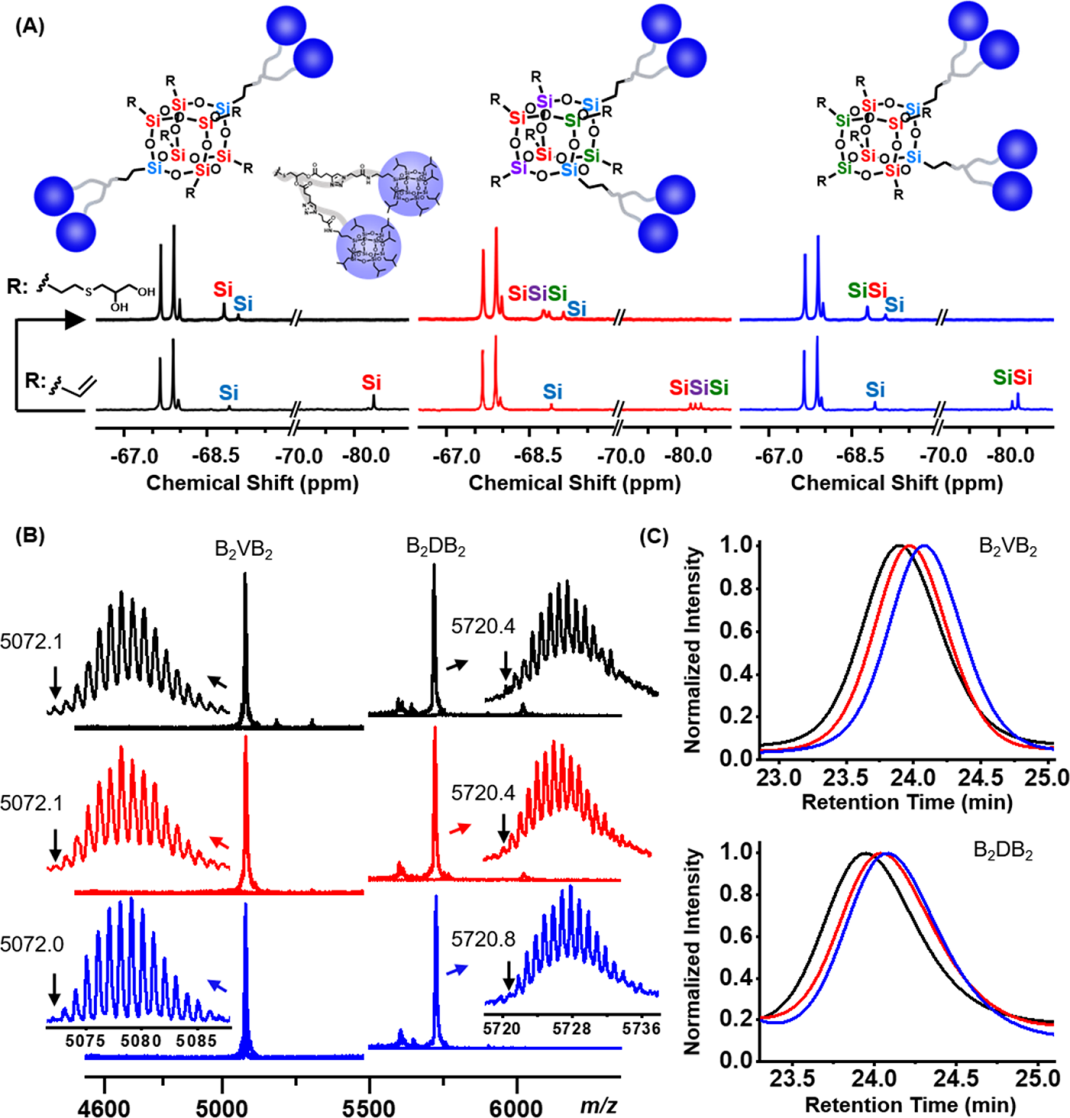
(A) ^29^Si NMR spectra show the conversion from B_2_VB_2_ (bottom) to B_2_DB_2_ (upper). (B) MALDI-TOF MS spectra show the monoisotopic peaks of B_2_VB_2_ (left) and B_2_DB_2_ (right). (C) SEC overlay of B_2_VB_2_ (upper) and B_2_DB_2_ (bottom). In all figures, black lines are used to denote the *para*-isomer, red lines the *meta*-isomer, and blue lines the *ortho*-isomer.

The evidence for each isomer’s regioconfiguration is from their ^29^Si NMR spectra (Figure 1A, bottom) where the resonances for the silicon atoms linked to the vinyl groups show up around −80 ppm and have only one peak (6 Si) for the *para*-isomer, three peaks (2:2:2) for the *meta*-isomer, and two peaks (2:4) for the *ortho*-isomer. After the final thiol–ene reaction, their chemical shifts move completely to −69 ppm with largely unchanged spectral patterns (Figure 1A, upper; and Figure S5).^25^Figure 1B shows the MALDI-TOF mass spectra of the intermediates during the synthesis. Although these isomers have identical molecular weights consistent with theoretical values, they have distinct retention volumes in size exclusion chromatography (SEC) (Figure 1C) with the *para*-isomer being the smallest, the *ortho*-isomer the largest, and the *meta*-isomer in between. Hence, the *para*-isomer has the most extended conformation, and the *ortho*-isomer has the most compact one. We can conclude that three isomers with precisely defined primary structures have been successfully synthesized.

### Characterization of Thermal Properties

**Figure 2:**
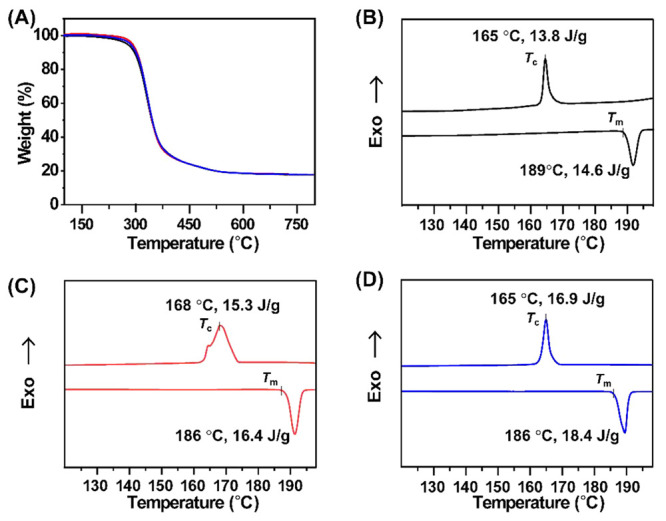
(A) TGA curves of the three isomers: black lines for *para*-, red lines for *meta*-, and blue lines for *ortho*-isomers. Second heating and first cooling differential scanning calorimetry traces of (B) *para*-, (C) *meta*-, and (D) *ortho*-isomers. The extrapolated onset temperatures are shown for the corresponding melting points, and the peak values are shown for crystallization.

**Figure 3:**
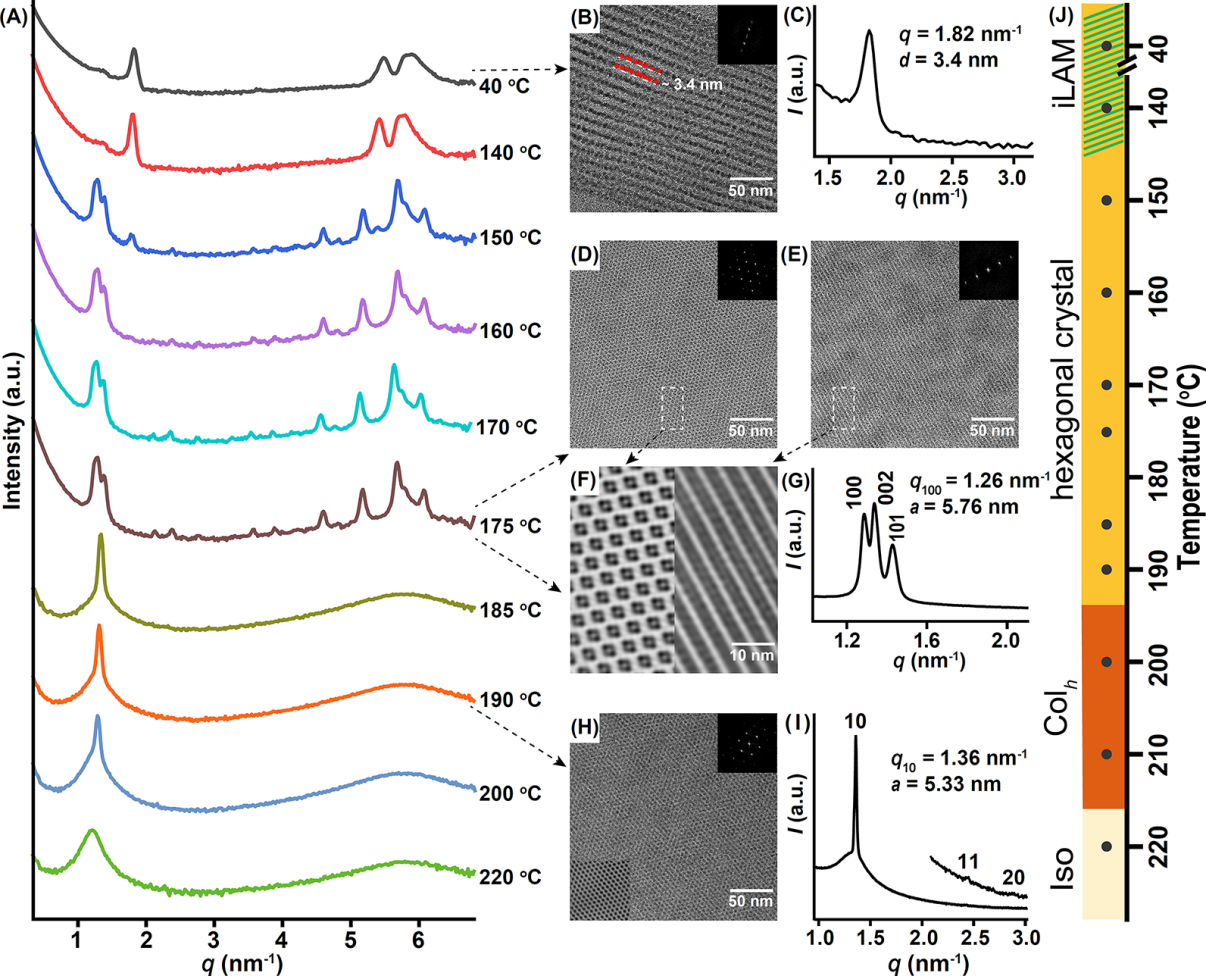
(A) TD-WAXS pattern of *para*-B_2_DB_2_ from 40 to 220 °C. TEM image (B) and SAXS curve (C) showing the ill-defined lamellar structure (iLAM) at 40 °C. TEM images (D–F) and SAXS curve (G) showing the crystal structure at 175 °C. TEM image (H) and SAXS curve (I) showing the microphase-separated Col_h_ phase at 185 °C. The top-right corner insets of TEM images (panels B, D, E, and H) are the FFT patterns. TEM images (F) and bottom-left corner insets of the TEM images (H) are images after the Fourier filtering. (J) Phase diagram corresponding to TD-WAXS. Iso: isotropic state.

**Figure 4:**
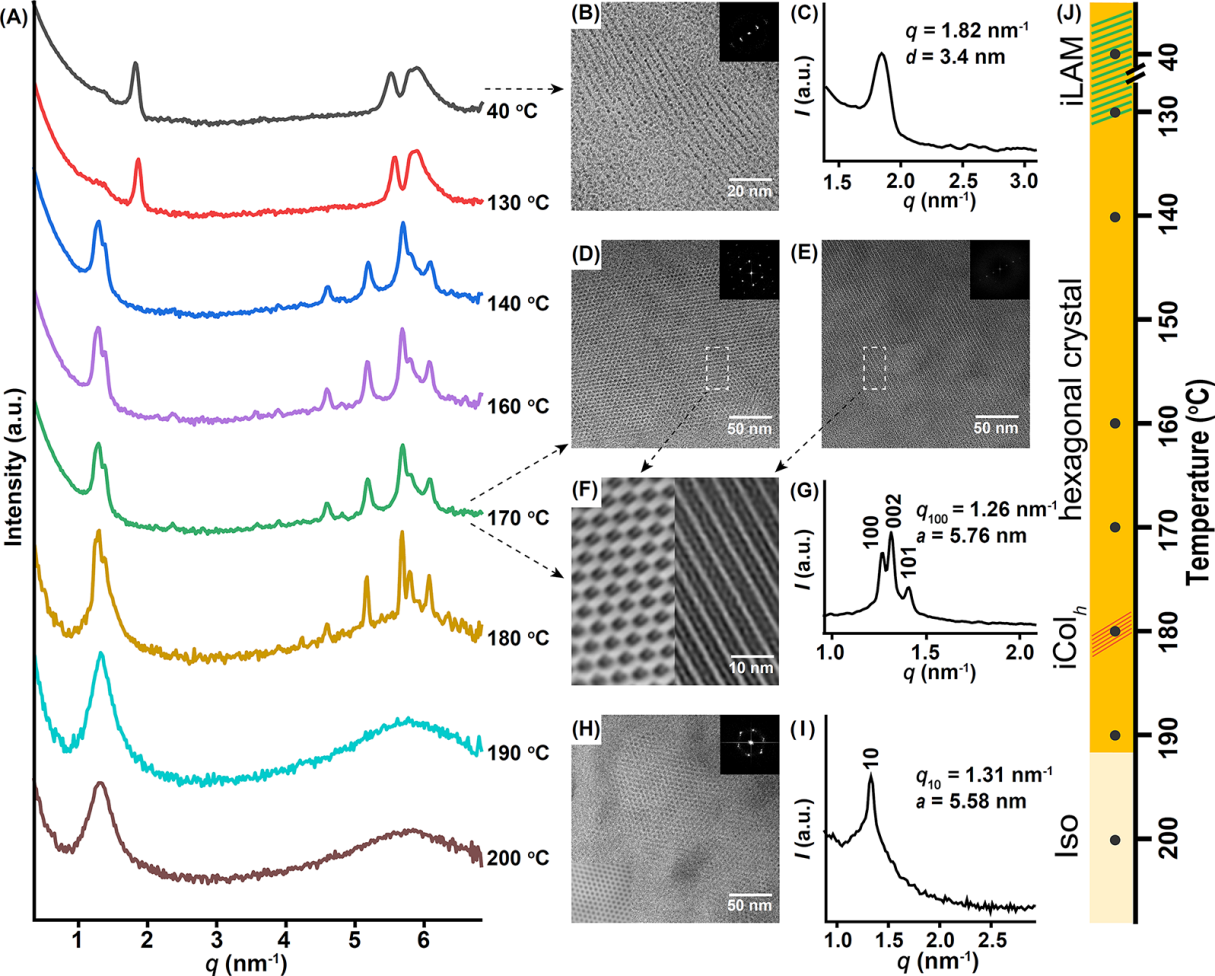
(A) TD-WAXS pattern of *meta*-B_2_DB_2_ from 40 to 200 °C. TEM image (B) and SAXS curve (C) showing the iLAM at 40 °C. TEM image (D–F) and SAXS curve (G) showing the hexagonal crystal developed at 170 °C. TEM image (H) and SAXS curve (I) showing the microphase-separated iCol_h_ phase at ∼180 °C after quenching and annealing. The top-right corner insets of TEM images in panels B, D, E, and H are the FFT patterns. The bottom-left corner insets of the TEM images in panels H and F are images after the Fourier filter. (J) Phase diagram corresponding to TD-WAXS. Iso: isotropic state.

**Figure 5:**
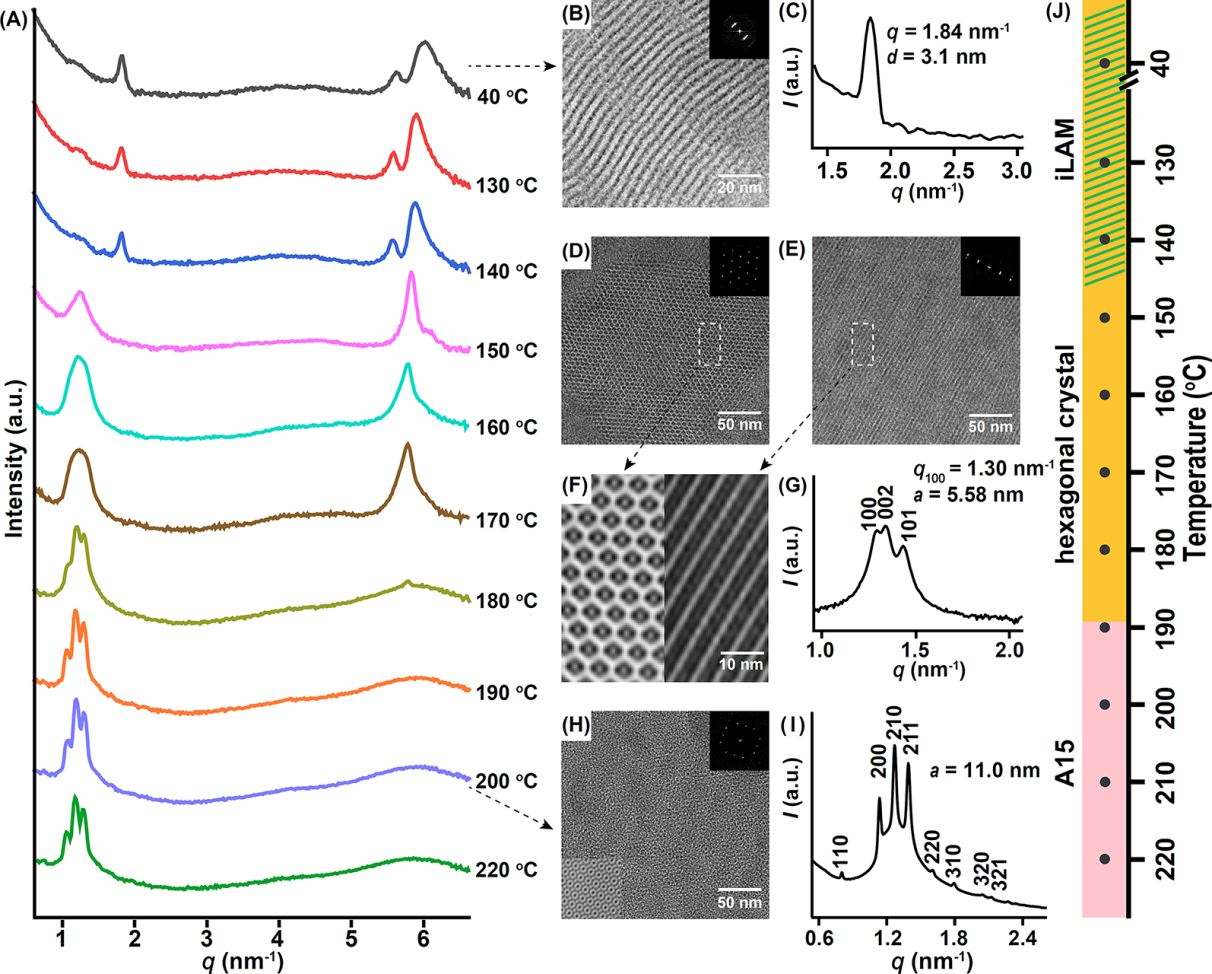
(A) TD-WAXS pattern of *ortho*-B_2_DB_2_ from 40 to 220 °C. TEM image (B) and SAXS curve (C) showing the ill-defined lamellae structure at 40 °C. TEM image (D–F) and SAXS curve (G) showing the superlattice after shearing and annealing. TEM image (H) and SAXS curve (I) showing the microphase-separated A15 phase after melting. The top-right corner insets of TEM images in panels B–E are the FFT pattern. The bottom-left corner insets of TEM images in panels H and F are images after the Fourier filter. (J) Phase diagram corresponding to TD-WAXS.

To facilitate structure development, the as-prepared samples were slowly evaporated from a THF/MeCN mixed solution (v/v = 1/1) and then dried in vacuo overnight at 60 °C before further characterization.^44^ To probe the phase behavior of these isomers, we evaluated their thermal stability using thermogravimetric analysis (TGA), which showed no obvious mass loss up to 200 °C (Figure 2A). In the differential scanning calorimetry (DSC) thermogram, the first cooling and second heating curves reveal similar melting temperatures (*T*_m_’s) and crystallization temperatures (*T*_c_’s) (Figure 2B–D). As summarized in Table 1, the *para*-B_2_DB_2_ has the highest *T*_m_ (189 °C), while *meta*- and *ortho*-B_2_DB_2_ have slightly lower *T*_m_’s (186 °C). There is considerable supercooling for crystallization (∼20 °C). All three isomers have similar *T*_c_’s (∼165 °C). Despite relatively simple DSC curves, we anticipated more complex phase behaviors for these samples. Hence, we followed the phase transitions of three isomers on heating using a combination of temperature-dependent wide-angle scattering (TD-WAXS), small-angle X-ray scattering (SAXS), and transmission electron microscopy (TEM) imaging (Figures 3–5), which indeed capture rich phase behaviors not obvious in the DSC.

### Phase Behaviors of the *para*-Isomer

For the *para*-isomer, the WAXS at 40 °C (Figure 3A) and the TEM images of the microtomed sample reveal a wavy lamellar morphology (Figure 3B). The layer spacing measured from the TEM image (∼3.4 nm) is consistent with the calculated value from SAXS (Figure 3C). This is assigned as an ill-defined, frustrated lamellae morphology (iLAM) whose formation is dominated by BPOSS crystallization.^44^ On heating above 150 °C, the metastable iLAM gradually transforms into a much ordered, stable crystalline phase, as evidenced by multiple sharp peaks in WAXS (Figure 3A). The SAXS profile of the sample annealed at 175 °C (Figure 3G) reveals a diffraction pattern with three sharp peaks. The full spectrum shown in Figure S6 suggests a hexagonal crystal structure with *a* = 5.76 nm, *c* = 9.59 nm.^71−73^ The corresponding index assignments are summarized in Table S1. To see the structure in real space, the crystalline sample was microtomed, stained, and imaged under TEM. As shown in Figure 3D–F, a large area of hexagonal honeycomb morphology (from ⟨001⟩ projection), as well as the layered morphology (from ⟨100⟩ projection), could be observed where the bright area is BPOSS, and the dark area is stained DPOSS. Selected area electron diffraction (SAED) shows the characteristic patterns of a hexagonal lattice (Figure S7) with dimensions matching those calculated from SAXS. The high-angle annular dark-field scanning transmission electron microscopy (HAADF-STEM) further shows a discernible contrast from the matrix to the core resembling the bright-field TEM image (Figure S8). Within this lattice, BPOSS crystallization is evidenced by the sharp diffraction peaks at ∼6.0 nm^–1^ (Figure S6) corresponding to the characteristic size of BPOSS (∼1.1 nm).^74^ We inferred that BPOSS would most likely crystallize into a matrix with a flat interface that wraps the core DPOSS cluster in a zigzag fashion.^44,75,76^ When the crystal melts at ∼185 °C, only one sharp peak remains in the small-angle region, indicating mesophase formation. This structure is determined to be a Col_h_ showing the characteristic (10), (11), and (20) lattice plane with the *q*/q* of 1, √3, and √4 from the synchrotron SAXS profile (Figure 3I). The calculated lattice dimension is 5.33 nm, slightly smaller than that of *a* in the crystalline phase. The TEM image of the sample quenched from 185 °C also demonstrates 6-fold symmetry (Figure 3H). On further heating, there is an order–disorder transition at ∼220 °C.

### Phase Behaviors of the *meta*-Isomer

For the *meta*-isomer, the WAXS at 40 °C (Figure 4A), the corresponding TEM image (Figure 4B), and SAXS profile (Figure 4C) reveal an iLAM morphology similar to that of the *para*-isomer with almost identical layer spacing (∼3.4 nm). On heating, crystallization occurs at ∼140 °C, leading to an ordered hexagonal crystal whose properties (see Figure 4D–F for TEM images, Figure 4G for the SAXS profile, and Table S2 for the index) are almost identical to those of the *para*-isomer. However, there is no mesophase formation on melting. The hexagonal crystal directly enters the isotropic state at ∼190 °C. We speculated that a mesophase similar to that in the *para*-isomer should also exist, albeit with limited stability, such that *T*_ODT_ is lower than the *T*_m_ of the hexagonal crystal. If so, since there is a ∼20 °C supercooling, it may be possible to develop such a mesophase during cooling before crystallization occurs. By quenching the sample from the isotropic state (200 °C) to 180 °C followed by annealing (Figure S9), an ordered phase was detected in SAXS, showing one sharp peak at *q* ∼ 1.3 nm^–1^ (Figure 4I). To capture this mesophase, the sample was immediately quenched in liquid nitrogen, microtomed, and stained for imaging under TEM. A faint pattern with 6-fold symmetry could be discerned in Figure 4H. Hence, we tentatively assign this phase to be an ill-defined Col_h_ (iCol_h_) mesophase with limited stability.

### Phase Behaviors of the *ortho*-Isomer

For the *ortho*-isomer, the sample also shows an iLAM morphology at 40 °C as revealed by WAXS (Figure 5A), TEM image (Figure 5B), and SAXS (Figure 5C). However, it does not develop into an ordered crystalline phase on heating. With the disappearance of the peak at 1.8 nm^–1^, only a broad peak at ∼1.3 nm^–1^ appears. We hypothesized that the flat interface required by crystallization may be incompatible with the cone shape of the *ortho*-isomer, making it difficult to develop highly ordered crystalline phases.^44,77^ On further heating above 170 °C where BPOSS crystallization no longer holds (Figure 5A), phase separation comes into play forming an ordered phase with *q*/q* equal to √2, √4, √5, √6, √8, , , , and  in the SAXS profile (Figure 5I). It is assigned as the Frank–Kasper A15 phase with a space group of *Pm*3*n*. The indexing is shown in Table S4, and the calculated lattice dimension is 11.07 nm. The A15 lattice was further confirmed by TEM imaging on microtomed samples. The corresponding fast Fourier transform (FFT) pattern and local TEM image after Fourier filtering are shown as insets of Figure 5H. An unambiguous 4^4^ tiling pattern along the ⟨100⟩ direction is characteristic of the A15 phase.^44^ The A15 phase is so stable that there is no order–disorder transition up to 220 °C.

Previously, it was found that mechanical shearing could help develop ordered structures.^78^ Thus, the sample was sheared at 188 °C and naturally cooled to room temperature. By doing so, the *ortho*-isomer formed a similar hexagonal crystal as the *para*- and *meta*-isomers, as revealed by TEM (Figure 5E–F) and SAXS (Figure 5G), with minor differences in lattice dimension (Table S3). Intriguingly, the sheared crystalline sample can hardly develop into A15 phase on melting (Figure S10). This could be understood based on the geometric mismatch of the two ordered structures. The *ortho*-isomer is cone-shaped with four BPOSS motifs pointing to the same side. Crystallization requires a flat interface between BPOSS motifs, while A15 phase is formed based on the stacking of supramolecular spherical aggregates favoring curved interfaces. The geometric mismatch means that their mutual transitions are hindered by a large kinetic barrier. The hexagonally packed molecules in the crystal could hardly rearrange into spherical aggregates for A15 phase formation. Similarly, cooling the A15 phases below the *T*_m_ would just lead to local BPOSS crystallization disrupting the A15 structure without global rearrangement into the hexagonal lattice (Figure S11).

### Phase Diagram of B_2_DB_2_ Isomers

**Figure 6:**
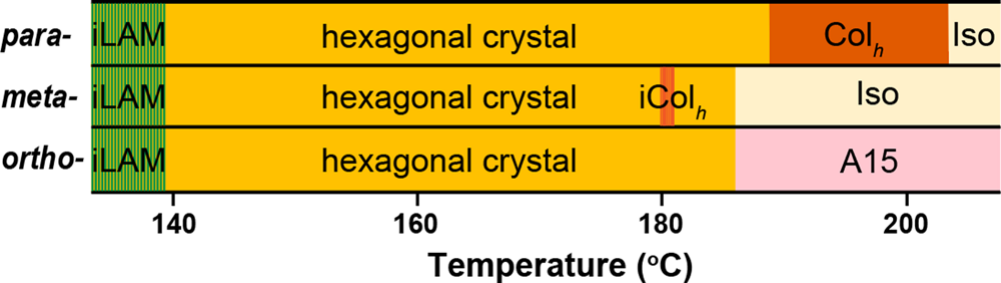
Phase diagram of B_2_DB_2_ regioisomers. The brown shaded lines in *meta*-isomer represent the metastable iCol_h_ phase; the green shaded lines at lower temperatures represent the metastable iLAM phase.

Based on the above discussions, we constructed phase diagrams for the three regioisomers (Figure 6). When evaporated from solution, three isomers adopt a kinetically trapped iLAM structure dominated by the BPOSS crystallization with a flat interface. Domination of BPOSS crystallization also occurs in solution where phase separation between BPOSS and DPOSS becomes trivial. Single-crystalline 2D nanosheets could be obtained for all three isomers from highly diluted solutions in DMF/toluene (Figure S12). The SAED pattern reveals that these nanosheets consisted of two layers of crystalline BPOSS and one layer of DPOSS.^70,79^ The formation of iLAM in bulk at low temperatures is thus not surprising. At elevated temperatures, molecules will reorganize to accommodate the BPOSS crystallization. When molecular symmetry allows it, as in *para*- and *meta*-isomers, formation of a globally ordered crystalline structure is spontaneous. Otherwise, it would require some assistance such as mechanical shearing to preorganize the molecules in the right orientation, as in the *ortho*-isomer. Nevertheless, they all form hexagonal crystals with minor differences in lattice dimension and molecular packing. Above *T*_m_, phase separation dominates the mesophase formation. The *para*-isomer forms Col_h_ which becomes isotropic at 220 °C while the *ortho*-isomer adopts a Frank–Kasper A15 phase stable even at 220 °C. As for the *meta*-isomer, an ill-defined, metastable iCol_h_ phase could only be developed within the narrow window of supercooling. The kinetics from the development of mesophases was very fast (less than 30 min) for all three isomers (Figure S13). With the only difference in regioconfiguration, these isomers indeed show rich phase behaviors.

### Proposed Molecular Packing Scheme

**Figure 7:**
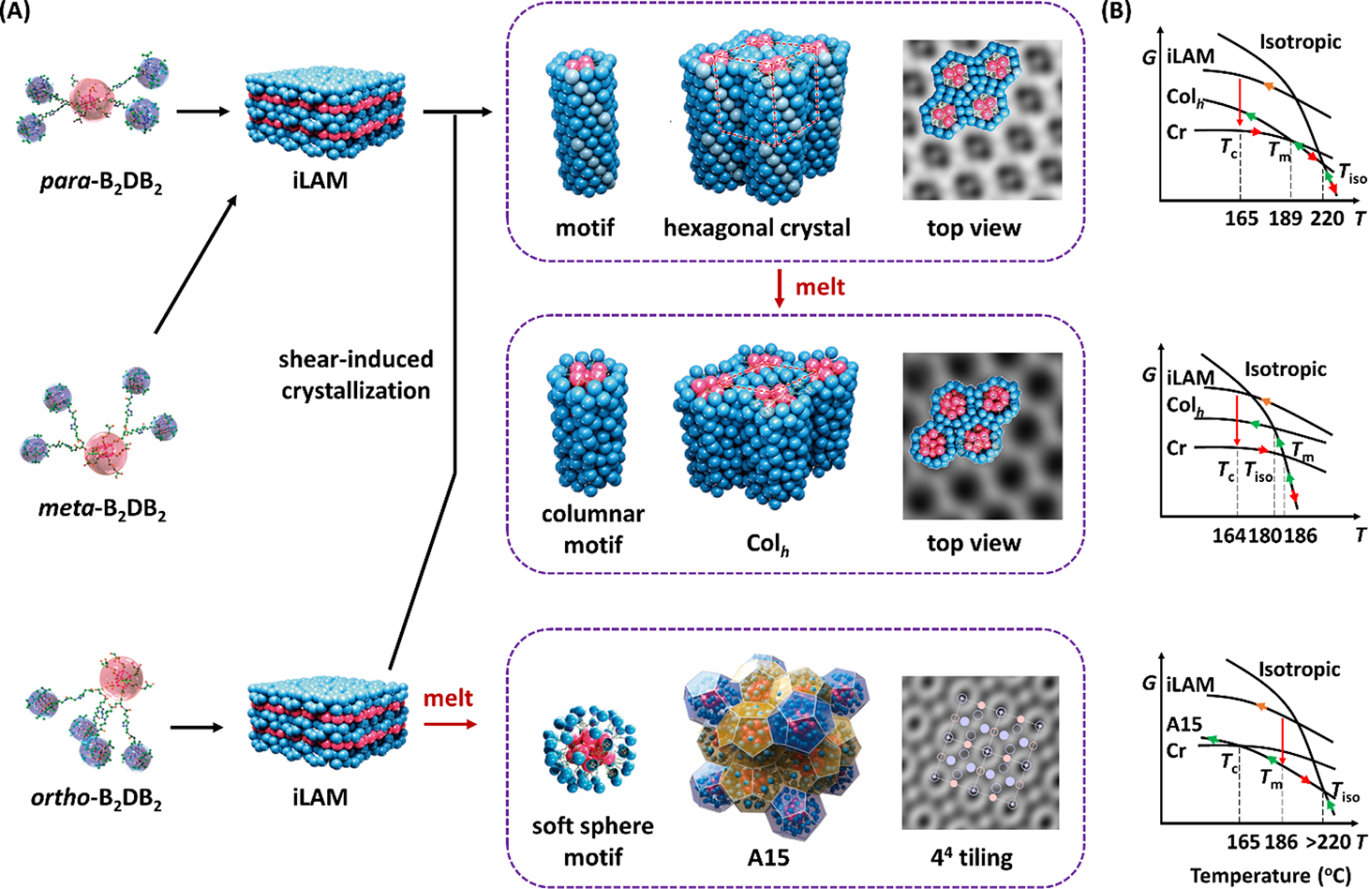
(A) Cartoon illustration of the typical phase structures and transition behavior of *para*-B_2_DB_2_, *meta*-B_2_DB_2_, and *ortho*-B_2_DB_2_ regioisomers. (B) Corresponding free energy profiles; red arrows represent the heating process, green arrows cooling processes with different protocols, and orange arrows the solution process.

To understand the phase diagram, we must understand the molecular packing in these structures. We assume that these structures may well be considered as “supramolecular crystals” possessing long-range translational order in terms of their basic self-assembled repeating units (motifs), but not necessarily in each atomic position within these motifs.^80^ Although the current data do not support structural resolution at the atomic level, we proposed the following molecular packing for ordered structures (Figure 7). On the molecular level, the *para*-isomer probably takes on an expanded conformation with tethered BPOSS motifs positioned on almost opposite sides, and the *ortho*-isomer likely adopts a cone shape with all four BPOSS motifs pointing to the same side, while the *meta*-isomer is somewhere in between. Considering the volume fraction of BPOSS, it is not surprising to see that, on melting, they undergo microphase separation to form mesophases with curved interfaces. Under this circumstance, the *para*- and *meta*-isomer may adopt a fan-shape conformation with four BPOSS units bending toward one side of DPOSS and forming a columnar structure (Figure 7A). From the lattice dimension of the Col_h_ mesophases, we could determine that there are ∼3 molecules within a 1 nm thick column for both isomers. The DPOSS core is probably not highly ordered and, thus, should be uniformly stained and show up as dark regions under TEM (Figures 3H and 4H). By contrast, the cone-shaped geometry of *ortho*-B_2_DB_2_ leads to spherical aggregate formation which further packs into an equilibrium A15 phase (Figure 7). On average, there are ∼22 molecules per spherical aggregate. At lower temperatures, BPOSS crystallization dominates, leading to formation of hexagonal crystals. The densities were determined to be ∼1.24 g/cm^3^ for the *para*- and *meta*-isomers and ∼1.22 g/cm^3^ for the *ortho*-isomer. From their lattice dimensions, we deduce that there are ∼18 molecules per unit cell for the *para*- and *meta*-isomers and ∼16.5 molecules for the *ortho*-isomer. Considering that the hexagonal crystal structure and Col_h_ mesophase are closely related, they may share a very similar packing scheme. It is likely that three molecules form a layer with DPOSS in the center and BPOSS in the periphery and that there are about six layers in a unit cell whose thickness is roughly consistent with the *c* axis dimension (∼9.59 nm). The central DPOSS should be highly ordered, and the peripheral BPOSS motifs are crystallized. Thus, staining mainly occurs at the side chain and linker region, but less on the central POSS core and the peripheral BPOSS layer. This explains the pattern observed under TEM where the central white dot is the unstained core of DPOSS, the zigzag-like white matrix the crystallized BPOSS motifs, and the region in between the heavily stained DPOSS side chains and linkers. From the current data, we could not pin down how BPOSS motifs are arranged within the unit cell. We speculate that they probably wrap around the central DPOSS in a helical pattern, which is supported by the TEM images (Figures 3F, 4F, and 5F, bright lines) along the *c*-direction showing faint traces of a spiral structure. Therefore, the configuration of the *para*-isomer is most compatible with this packing, and the corresponding crystal is the easiest to form with the highest stability. The geometric mismatch between the *meta*-isomer configuration and the hexagonal crystal would introduce considerable tension on the linker region, which destabilizes the crystal as shown by its lower *T*_m_. It is an even bigger challenge to accommodate the cone-shaped *ortho*-isomer into this packing scheme. As a result, the hexagonal crystal of the *ortho*-isomer is the most difficult one to form, requiring the assistance of mechanical shearing. Deformation of the unit cell also occurs which can accommodate fewer molecules per unit cell (∼16.5). Due to lattice symmetry mismatch, the transition between the hexagonal crystal and A15 mesophase is slow with a large kinetic barrier (Figure S10). The A15 phase could only be developed either by annealing a noncrystalline sample through an ill-defined lamellar intermediate (Figure 5A) or by quenching from the isotropic state (Figure S13C).

### Understanding Phase Stability

The stabilities of the mesophase and crystal are evaluated by *T*_iso_ and *T*_m_, respectively. The mesophase stability follows the order of A15 (*ortho*) > Col_h_ (*para*) > iCol_h_ (*meta*). Since the mesophase stability is critically related to the average cross-section area per molecule (*A*_0_, see Table 1 and Table S5), we compared the mesophases and the corresponding *A*_0_ values for the regioisomers of BDB,^24^ B_2_DB_2_, DPOSS-2PS (or SDS),^21^ and S_2_DS_2_^22^ with similar volume fractions of the hydrophobic matrix. For consistency, the linker region was counted as part of the hydrophobic matrix, and thus, some of the data were recalculated based on the reported values. The calculated *A*_0_ is ∼2.21 nm^2^ for the Col_h_ mesophase formed by the *para*-isomer (eq S6) and ∼2.16 nm^2^ on average for the A15 mesophase formed by the *ortho*-isomer (eq S7). The higher stability of the A15 phase is reflected in its small *A*_0_ values, which is probably caused by a significantly larger number of molecules in one soft sphere that collectively stabilized the structure.

By comparing similar isomers in this series of samples (Table S5), we can see the following trends: (1) When the tethered part is the particulate BPOSS, the formation of ordered structures will be intricately dependent on the core geometry, which is easier in the cases of *para*- or *ortho*-isomers when the geometry matches but is rather difficult for the *meta*-isomer. (2) When the ordered structures can form in BDB and B_2_DB_2_, they tend to be more stable than the counterparts with tethered PS chains, which can be rationalized by the corresponding smaller entropic change in order–disorder transition. (3) For similar molecular geometries, compared to PS, tethering a BPOSS particle increases the conformational asymmetry at the interface and promotes the formation of curved morphologies and unconventional spherical phases at comparable or smaller volume fractions. (4) In all cases, the *para*-isomer seems to possess the highest *A*_0_ values, and the *ortho*-isomer tends to have the smallest *A*_0_ values, suggesting that the *ortho*-isomer has the most stable phase-separated structure among the three isomers since the clustered geometry is preorganized for phase separation. These results improve our understanding on the effect of macromolecular isomerism on the phase behaviors of giant molecule assemblies.

The introduction of the BPOSS motif makes crystallization a strong competing interaction to phase separation. When the two act in synergy, the structure stability is enhanced. For all samples, the hexagonal crystal is the thermodynamically stable phase, and the iLAM is metastable at lower temperatures. The *para*-isomer has the highest *T*_m_ for the crystal because its symmetry is most compatible with the proposed packing, whereas the other two have comparably smaller *T*_m_’s. These competing interactions are also the origin of metastable phases and monotropic phase behaviors. To rationalize the phase behavior of these isomers, we demonstrate the transition pathways in the postulated free energy landscape in Figure 7B. As for *para*- and *ortho*-B_2_DB_2_, the Col_h_ and A15 are equilibrium phases. For *meta*-B_2_DB_2_, the iCol_h_ phase is metastable, with its isotropic temperature (*T*_iso_) lower than its melting point *T*_m_. Compared to previous results on the self-assembly of DPOSS-BPOSS molecular systems,^44,46^ the reduced number of hydroxyl groups (from 14 to 12) leads to a subtle change in the free energy landscape. Together with the regioconfiguration, considerable phase complexity is generated. We envision that these kinds of Janus nanograins with tunable phase structures and sophisticated phase behaviors can serve as precursors in the fabrication of mesoporous nanosilica materials for catalysis, separation, etc.^81−83^

## Conclusion

In summary, a series of precisely defined regioisomeric Janus nanograins were synthesized and found to exhibit complex phase behaviors, forming distinct nanostructures as a combined result of symmetry/geometry, phase separation, and crystallization. When properly crystallized, three isomers form very similar hexagonal crystals. Notably, the *ortho*-isomer requires mechanical shearing to overcome the high nucleation barrier for ordered packing. On melting, phase separation dominates the mesophase formation. The Col_h_ phase and Frank–Kasper A15 phase were further detected in *para*- and *ortho*-isomers, while the *meta*-isomer exhibited a metastable and ill-defined Col_h_ phase due to the mismatched geometry. Also due to geometry mismatch, the transition between hexagonal crystal and Frank–Kasper A15 phase in the *ortho*-isomer was found to be very slow. Moreover, the stability of these phases was in general agreement with previous findings in counterparts, with the *para*-isomer having the most stable crystal structure and the *ortho*-isomer having the most stable mesophase. The results highlight a profound influence of minute structural changes on the assembly outcomes and suggest that regioconfiguration provides a new dimension for generating phase complexity and fine-tuning the structures and properties of materials. This affords enticing opportunities to comprehensively assess the role of macromolecular isomerism in the self-assembly of nanoscaled amphiphilic giant molecules and to leverage isomerism for controlled phase complexity and delicate function in soft matter.

## Supporting Information Available

Chemicals and instrumentation, experimental procedures, density measurement, detailed characterizations, and calculations (PDF)
