# Supplementary material for: Leveraging Macromolecular Isomerism for Phase Complexity in Janus Nanograins
Source: ACS Cent Sci. 2023 Feb 8;9(2):289–99. doi: 10.1021/acscentsci.2c01405 (PMC9951285; doi:10.1021/acscentsci.2c01405)
Supplement: Supplementary file 1 — oc2c01405_si_001.pdf [file oc2c01405_si_001.pdf]

Supporting information

for

Leveraging Macromolecular Isomerism for Phase Complexity in Janus Nanograins

Yu Shao,[1,†] Di Han,[2,†] Yangdan Tao,[2] Fengfeng Feng,[3] Ge Han,[2] Bo Hou,[1] Hao Liu,[3] Shuguang Yang,[3] Qiang Fu[2,] Wen-Bin Zhang,[1,*]*

[1] Beijing National Laboratory for Molecular Sciences, Key Laboratory of Polymer Chemistry & Physics of Ministry of Education, College of Chemistry and Molecular Engineering, Center for Soft Matter Science and Engineering, Peking University, Beijing 100871, China

[2] College of Polymer Science & Engineering, State Key Laboratory of Polymer Materials Engineering, Sichuan University, Chengdu 610065, China

[3] State Key Laboratory for Modification of Chemical Fibers and Polymer Materials, Center for Advanced Low-Dimension Materials, Donghua University, Shanghai 201620, China

E-mail: qiangfu@scu.edu.cn (Q. F.); wenbin@pku.edu.cn (W.-B. Z.)

† These authors contribute equally to this work.

Experimental section

Chemicals and Solvents. Octavinyl-POSS (T_8V_8, 98%, Beijing HWRK Chemical Co., LTD), BPOSS-NH_2 (Hybrid Plastics, AM0265), 1-thioglycerol (J&K Chemicals, 95%), 2,2-dimethoxy-2-phenylacetophenone (DMPA, J&K Chemical, 98%), 4-pentynoic acid (J&K Chemicals, 95%), N, N'-diisopropyl carbodiimide (DIPC, J&K Chemicals, 99%), 4-dimethylaminopyridine (DMAP, J&K Chemicals, 99%), 3-bromopropionic acid (J&K Chemicals, 98%), Sodium azide (NaN_3, Alfa Aesar, 99%), 2-hydroxy-4'-(2-hydroxyethoxy)-2-methylpropiophenone (Irgacure 2959, J&K Chemicals, 98%), *N, N, N',N'',N''*-pentamethyl diethylenetriamine (PMDETA, J&K Chemicals, 98%), cuprous bromide (CuBr, J&K Chemicals, 98%) was purified by washing with acetic acid for three times, washed with methanol, and dried in vacuum. Sodium chloride (NaCl), sodium sulfate (Na_2SO_4), celite, petroleum ether (PE, b.p. 60-90 °C), ethyl acetate (EA), methanol (MeOH), dichloromethane (DCM), and tetrahydrofuran (THF) were purchased from Chengdu Kelong Chemical Co., Ltd. All the solvents were used after distillation or dried over a solvent purification system (SPS-5, Etelux, Beijing, China). BPOSS-N_3 used in this work was prepared according to our previous report.[1] The synthesis of V_6T_8-4yne regioisomers is detailed in the reference.[2]

Instrumentation and Characterizations.

The UV lamp used in this work is Rayonet RPR-200 Photochemical Reactor with RPR-3500A lamp (3W*12, λ = 350 nm, USA). ^{1}H NMR spectra were obtained in $CDCl_3$ by using a Bruker 400 MHz NMR spectrometer, ^{13}C and ^{29}Si NMR spectra were obtained in $CDCl_3$ by using a Bruker 500 MHz NMR spectrometer. FT-IR spectrometer was carried out on a Nicolet 6700 spectrometer (Nicolet, U.S.), and the samples were grinded with KBr before scanned. Thermogravimetric analysis (TGA) was performed on a thermo-analyzer instrument (TA Instruments Inc., USA) with a scan rate of 10 °C min^{-1} under nitrogen atmosphere. The differential scanning calorimetry (DSC) was carried out by

using a TA Instruments under nitrogen atmosphere with a heating rate of 10 °C min^{-1} to obtain their melting temperature and the crystallization temperature. Size exclusion chromatography (SEC) results were performed on a Viscoteck SEC 270maxTM (Malvern, U.K.). The samples were dissolved in THF with the concentration of 2 mg mL^{-1} and THF was also used as eluent (flow rate: 0.10 mL min^{-1}, pump pressure: 6 Pa). Matrix-assisted laser desorption ionization time-of-flight (MALDI-TOF) mass spectra were performed on a MALDI TOF/TOF 5800 mass spectrometer (AB Sciex, USA). A positive reflection mode was used with *trans*-2-[3-(4-tert-butylphenyl)-2-methyl-2-propenylidene]malononitrile (DCTB, TCI, >99%) as the matrix and was dissolved in $CHCl_3$ (20 mg mL^{-1}). Sodium trifluoromethanesulfonate (NaTFA, J&K Chemicals, 98%,) was dissolved in a mixture of MeOH/$CHCl_3$ (v/v = 1/3) at a concentration of 10 mg mL^{-1}, which served as the cationizing agent solution. The samples were typically dissolved in $CHCl_3$ with the concentration of 5 mg mL^{-1}. To prepare the measured samples, 0.5 μL of matrix and the cationizing agent solution mixture was deposited on the wells of a 384-well ground-steel plate and allowed the spots to dry, then depositing 0.5 μL of each sample on top of the spot of dry matrix, finally deposition of another 0.5 μL of matrix and the cationizing agent solution mixture on top of the dry sample. Small/Wide-Angle X-ray Scattering (SAXS/WAXS) experiments were recorded at beamline BL16B1 of the Shanghai Synchrotron Radiation Facility (SSRF), beamline 1W2A of the Beijing Synchrotron Radiation Facility (BSRF), and Ganesha SAXS Lab at Peking University. Silver behenate (with the *d*-spacing of 58.38Å) was used for the calibration of sample to detector distance. Bright field transmission electron microscope (TEM) images of the thin-slice samples were recorded on a field emission transmission electron microscopy (FEI Tecnai F20, USA) with an accelerating voltage of 200 kV on a digital CCD camera. High-angle annular dark-field scanning transmission electron microscopy (HAADF-STEM) images and selected area electron diffraction (SAED) experiments were recoded using the same

equipment.

Sample Preparation

Crystalline samples of B_2DB_2 for X-ray experiments were prepared by first dissolving about 100 mg samples in a THF/MeCN (v/v = 1/1) mixed solvent. The solution was then allowed to slowly evaporate at room temperature to give bulk powder samples, which were further dried in vacuo overnight at 60°C. The dried powder samples were sealed into an aluminum sample holder with a hole of 2 mm diameter for SAXS and WAXS measurements. Powder samples treated by the same procedure were also transferred to an aluminum DSC pan with a weight of about 2 mg for DSC measurements.

To prepare thermally annealed samples at higher temperatures, *para*- and *ortho*-isomer were directly annealed at 190 °C for 2 h, *meta*-isomer was first heated to 200 °C with a heating rate of 30 °C/min, then followed by immediate cooling to 178~182 °C and then annealed for 0.5-24 h. All thermal annealing procedures were performed under vacuum. After cooled to room temperature, the samples were used in SAXS and WAXS measurements. Noted that once the mesophases were formed (Col_h for *para*-, $iCol_h$ for *meta*-, and A15 for *ortho*-isomers), slowly cooled to room temperature or quenched in liquid nitrogen will form the same structure. At this stage, phase separation will dominate the structure, the crystallization of BPOSS is restrained.

Once the ordered structures were formed, they were immediately quenched into the liquid nitrogen and microtomed for transmission electron microscopy (TEM) observations. Thin slices of the bulk samples suitable for TEM experiments were obtained using a Reichert Ultracut S (Leica) microtome on annealed samples embedded in epoxy monolith at room temperature. The slices were transferred to carbon coated copper grids for TEM experiments. The thickness of these thin slices was around 70-100 nm. When necessary, staining of the samples was performed at room temperature by using RuO_4 for 10 min. After BF TEM images were obtained, the experimental SAED patterns were

then taken at the same area of the microtomed sample. The *d*-spacings were calibrated using a TlCl standard.

Synthetic Procedures and Molecular Characterizations.

Synthesis of 2BPOSS-VPOSS-2BPOSS (B_2VB_2). In a glove box, V_6T_8-4yne (100 mg, 0.09 mmol, 1 equiv), BPOSS-N_3 (400 mg, 0.41 mmol, 4.8 equiv), CuBr (3 mg) were added into a 50 mL Schlenk flask with a magnetic stirring bar and dissolved in 10 mL anhydrous THF. After that, PMDETA (30 mg, 36 μL) was added via pipette. And then, the Schlenk flask was sealed and stirred at room temperature for 24 h. The mixture was purified by flash column chromatography on the silica gel with DCM/EA (v/v = 2:1) as eluent to afford a white powder.

***para*-B_2VB_2.** Yield: 70%. ^{1}H NMR ($CDCl_3$, 400 MHz, ppm): δ = 7.48 (s, 4H, triazole), 6.17-5.84 (m, 18H, vinyl-H), 5.11 (m, 2H, -$CH_2C\underline{H}$(OCO-)CH_2-), 4.62 (m, 8H, -$C\underline{H}_2$-N), 4.39-4.10 (m, 4H, -CH_2CH(OCO-)$C\underline{H}_2$-), 3.21 (m, 8H, -NH-$C\underline{H}_2$-), 2.97 (t, 8H, -$CH_2C\underline{H}_2$-C(triazole)), 2.84-2.59 (m, 24H, -$C\underline{H}_2SC\underline{H}_2$, -OOC-$C\underline{H}_2$-, -$C\underline{H}_2$-CONH-), 1.93-1.77 (m, 28H, -$CH_2C\underline{H}(CH_3)_2$), 1.64-1.50 (m, 8H, -$C\underline{H}_2CH_2Si$ (BPOSS)), 0.95 (dd, 168H, -$CH_2CH(C\underline{H}_3)_2$), 0.60 (d, 68H, -$C\underline{H}_2Si$). ^{13}C NMR ($CDCl_3$, 125 MHz, ppm): δ = 172.19, 171.81, 145.77, 137.21, 128.51, 122.58, 70.55, 63.83, 46.13, 42.05, 36.41, 33.42, 33.21, 31.91, 26.73, 25,70, 25.69, 23.88, 23.85, 22.89, 22.48, 22.44, 20.74, 20.68, 13.04, 9.48. ^{29}Si NMR (99 MHz, $CDCl_3$, ppm): δ = -67.64, -67.87, -67.97, -68.87, -80.34. MALDI-TOF MS: calcd monoisotopic mass for $[M{\cdot}Na]^+$ ($C_{178}H_{352}N_{16}NaO_{72}S_2Si_{40}$) = 5072.4 Da. Found: 5072.0 Da.

***meta*-B_2VB_2.** Yield: 72%. ^{1}H NMR ($CDCl_3$, 400 MHz, ppm): δ = 7.48 (s, 4H, triazole), 6.17-5.84 (m, 18H, vinyl-H), 5.12 (m, 2H, -$CH_2C\underline{H}$(OCO-)CH_2-), 4.62 (m, 8H, -$C\underline{H}_2$-N), 4.39-4.10 (m, 4H, -CH_2CH(OCO-)$C\underline{H}_2$-), 3.18 (m, 8H, -NH-$C\underline{H}_2$-), 2.97 (t, 8H, -$CH_2C\underline{H}_2$-C(triazole)), 2.84-2.59 (m, 24H, -$C\underline{H}_2SC\underline{H}_2$, -OOC-$C\underline{H}_2$-, -$C\underline{H}_2$-CONH-), 1.93-1.77 (m, 28H, -$CH_2C\underline{H}(CH_3)_2$), 1.64-1.50 (m, 8H, -$C\underline{H}_2CH_2Si$(BPOSS)), 0.95 (dd, 168H, -$CH_2CH(C\underline{H}_3)_2$), 0.60 (d, 68H, -$C\underline{H}_2Si$). ^{13}C NMR ($CDCl_3$, 125

MHz, ppm): δ = 172.19, 171.82, 145.77, 137.24, 137.17, 128.54, 122.55, 70.55, 63.85, 46.12, 42.06, 36.41, 33.42, 33.22, 31.90, 26.72, 25,70, 25.69, 23.88, 23.85, 22.90, 22.48, 22.44, 20.75, 20.69, 13.03, 9.49. ^{29}Si NMR (99 MHz, $CDCl_3$, ppm): δ = -67.64, -67.87, -67.96, -68.87, -80.25, -80.33, -80.43. MALDI-TOF MS: calcd monoisotopic mass for $[M{\cdot}Na]^+$ ($C_{178}H_{352}N_{16}NaO_{72}S_2Si_{40}$) = 5072.4 Da. Found: 5072.2 Da.

***ortho*-B$_2$VB$_2$.** Yield: 69%. ^{1}H NMR ($CDCl_3$, 400 MHz, Hz, ppm): δ = 7.51 (dd, 4H, triazole), 6.17-5.84 (m, 18H, vinyl-H), 5.11 (m, 2H, $-CH_2C\underline{H}(OCO-)CH_2-$), 4.62 (m, 8H, $-C\underline{H}_2-N$), 4.39-4.11 (m, 4H, $-CH_2CH(OCO-)C\underline{H}_2-$), 3.19 (m, 8H, $-NH-C\underline{H}_2-$), 2.99 (t, 8H, $-CH_2C\underline{H}_2-C$(triazole)), 2.84-2.59 (m, 24H, $-C\underline{H}_2SC\underline{H}_2$, $-OOC-C\underline{H}_2-$, $-C\underline{H}_2-CONH-$), 1.93-1.77 (m, 28H, $-CH_2C\underline{H}(CH_3)_2$), 1.64-1.50 (m, 8H, $-C\underline{H}_2CH_2Si$(BPOSS)), 0.95 (dd, 168H, $-CH_2CH(C\underline{H}_3)_2$), 0.60 (d, 68H, $-C\underline{H}_2Si$). ^{13}C NMR ($CDCl_3$, 125 MHz, ppm): δ = 172.18, 171.81, 145.74, 137.20, 137.12, 128.53, 122.61, 70.53, 63.87, 46.17, 42.07, 36.40, 33.41, 33.20, 31.90, 26.68, 25,70, 25.69, 23.88, 23.85, 22.90, 22.48, 22.44, 20.73, 20.67, 13.00, 9.50. ^{29}Si NMR (99 MHz, $CDCl_3$, ppm): δ = -67.64, -67.88, -67.95, -68.90, -80.24, -80.34. MALDI-TOF MS: calcd monoisotopic mass for $[M{\cdot}Na]^+$ ($C_{178}H_{352}N_{16}NaO_{72}S_2Si_{40}$) = 5072.4 Da. Found: 5072.1 Da.

Synthesis of B$_2$DB$_2$ isomers. In a 5 mL vial with a magnetic stirring bar, B$_2$VB$_2$ (150 mg, 0.03 mmol, 1 equiv), 1-thioglycerol (39 mg, 0.36 mmol, 12 equiv), and the photoinitiator Irgacure 2959 (3 mg) were fully dissolved in 3 mL THF. The mixture was reacted under irradiation by using UV light (365 nm) for 20 min. The solution mixture was purified by precipitation into the mixture of water/methanol (v/v = 1:1) for three times to afford a white powder.

***para*-B$_2$DB$_2$.** Yield: 68%. ^{1}H NMR ($CDCl_3$, 400 MHz, ppm): δ = 7.59 (s, 4H, triazole), 6.11 (s, 1H, -NH-), 5.13 (m, 2H, $-CH_2C\underline{H}(OCO-)CH_2-$), 4.64 (t, 8H, $-C\underline{H}_2-N$), 4.38-4.11 (m, 4H, $-CH_2CH(OCO-)C\underline{H}_2-$), 3.92-3.44 (m, 30H), 3.20 (m, 8H, $-NH-C\underline{H}_2-$), 3.01 (m, 8H, $-CH_2C\underline{H}_2-$

C(triazole)), 2.90-2.55 (m, 48H, -CH(OH)$C\underline{H}_2SC\underline{H}_2$,-$C\underline{H}_2SC\underline{H}_2$, -OOC-$C\underline{H}_2$-, -$C\underline{H}_2$-CONH-), 1.84 (m, 28H, -$CH_2C\underline{H}(CH_3)_2$), 1.57 (m, 8H, -$C\underline{H}_2CH_2Si$(BPOSS)), 1.26 (m, 16H, -$CH_2SCH_2C\underline{H}_2Si$-), 0.95 (dd, 168H, -$CH_2CH(C\underline{H}_3)_2$), 0.60 (m, 68H, -$C\underline{H}_2Si$-). ^{13}C NMR ($CDCl_3$, 125 MHz, ppm): δ = 172.24, 169.17, 145.62, 122.79, 70.95, 69.26, 65.44, 63.96, 46.17, 42.16, 36.39, 31.91, 29.53, 29.32, 25.70, 23.86, 22.87, 22.46, 20.69, 14.13, 9.51. ^{29}Si NMR (99 MHz, $CDCl_3$, ppm): δ = -67.62, -67.65, -67.89, -67.99, -68.72, -68.78, -69.03. MALDI-TOF MS: calcd monoisotopic mass for $[M{\cdot}Na]^+$ $C_{196}H_{400}N_{16}NaO_{84}S_8Si_{40}$:5720.6, found: 5720.4.

***meta*-B$_2$DB$_2$.** Yield: 71%. ^1H NMR ($CDCl_3$, 400 MHz, ppm): δ = 7.63 (s, 4H, triazole), 6.10 s, 1H, -NH-), 5.14 (m, 2H, -$CH_2C\underline{H}$(OCO-)CH_2-), 4.65 (t, 8H, -$C\underline{H}_2$-N), 4.39-4.10 (m, 4H, -CH_2CH(OCO-)$C\underline{H}_2$-), 3.94-3.50 (m, 30H), 3.19 (m, 8H, -NH-$C\underline{H}_2$-), 3.02 (m, 8H, -$CH_2C\underline{H}_2$-C(triazole)), 2.93-2.57 (m, 48H, -CH(OH)$C\underline{H}_2SC\underline{H}_2$,-$C\underline{H}_2SC\underline{H}_2$, -OOC-$C\underline{H}_2$-, -$C\underline{H}_2$-CONH-), 1.84 (m, 28H, -$CH_2C\underline{H}(CH_3)_2$), 1.56 (m, 8H, -$C\underline{H}_2CH_2Si$ (BPOSS)), 1.25 (m, 16H, -$CH_2SCH_2C\underline{H}_2Si$-), 0.94 (dd, 168H, -$CH_2CH(C\underline{H}_3)_2$), 0.59 (m, 68H, -$C\underline{H}_2Si$-). ^{13}C NMR ($CDCl_3$, 125 MHz, ppm): δ = 172.27, 169.20, 145.90, 122.75, 71.07, 70.48, 65.45, 63.94, 47.25, 42.14, 36.38, 33.24, 31.93, 29.70, 26.72, 25.70, 23.86, 22.88, 22.46, 21.94, 20.71, 14.13, 12.83, 9.52. ^{29}Si NMR (99 MHz, $CDCl_3$, ppm): δ = -67.65, -67.89, -67.98, -68.72, -68.74, -68.83, -69.08. MALDI-TOF MS: calcd monoisotopic mass for $[M{\cdot}Na]^+$ $C_{196}H_{400}N_{16}NaO_{84}S_8Si_{40}$:5720.6, found: 5720.4.

***ortho*-B$_2$DB$_2$.** Yield: 66%. ^1H NMR ($CDCl_3$, 400 MHz, ppm): δ = 7.60 (s, 4H, triazole), 6.10 (s, 1H, -NH-), 5.13 (m, 2H, -$CH_2C\underline{H}$(OCO-)CH_2-), 4.64 (t, 8H, -$C\underline{H}_2$-N), 4.39-4.10 (m, 4H, -CH_2CH(OCO-)$C\underline{H}_2$-), 3.94-3.50 (m, 30H), 3.20 (m, 8H, -NH-$C\underline{H}_2$-), 3.01 (m, 8H, -$CH_2C\underline{H}_2$-C (triazole)), 2.93-2.57 (m, 48H, -CH(OH)$C\underline{H}_2SC\underline{H}_2$,-$C\underline{H}_2SC\underline{H}_2$, -OOC-$C\underline{H}_2$-, -$C\underline{H}_2$-CONH-), 1.85 (m, 28H, -$CH_2C\underline{H}(CH_3)_2$), 1.57 (m, 8H, -$C\underline{H}_2CH_2Si$ (BPOSS)), 1.26 (m, 16H, -$CH_2SCH_2C\underline{H}_2Si$-), 0.95 (dd, 168H, -$CH_2CH(C\underline{H}_3)_2$), 0.60 (m, 68H, -$C\underline{H}_2Si$-). ^{13}C NMR ($CDCl_3$, 125 MHz, ppm): δ = 172.25,

169.19, 145.81, 122.70, 71.09, 70.48, 65.40, 63.96, 46.16, 42.13, 36.37, 33.35, 31.91, 29.70, 25.70, 23.86, 22.88, 22.46, 20.71, 14.13, 12.76, 9.52. ^{29}Si NMR (99 MHz, $CDCl_3$, ppm): δ = -67.66, -67.89, -67.98, -68.76, -69.08. MALDI-TOF MS: calcd monoisotopic mass for $[M{\cdot}Na]^+$ $C_{196}H_{400}N_{16}NaO_{84}S_8Si_{40}$:5720.6, found: 5720.8.

Section I. Molecular Characterizations.

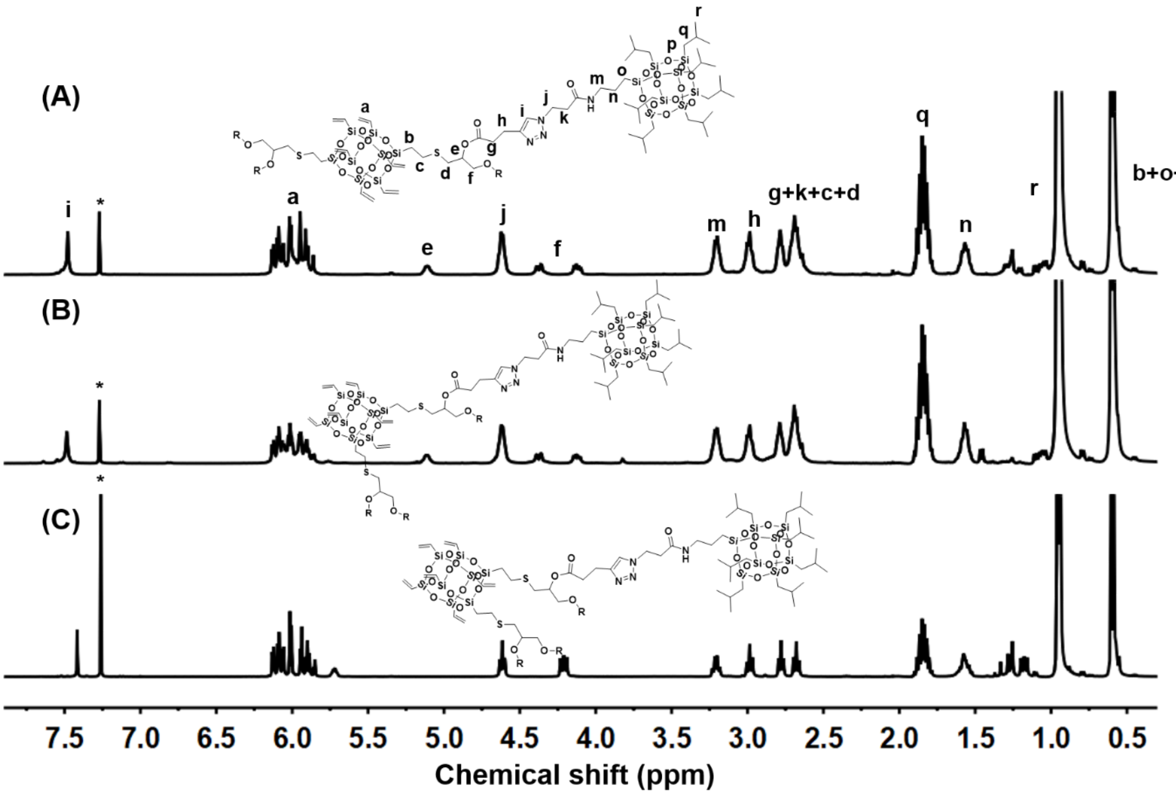

Figure S1. ^{1}H NMR spectra of (A) *para*-B_2VB_2, (B) *meta*-B_2VB_2, and (C) *ortho*-B_2VB_2. Asterisks at 7.27 ppm are resonances from residual $CHCl_3$.

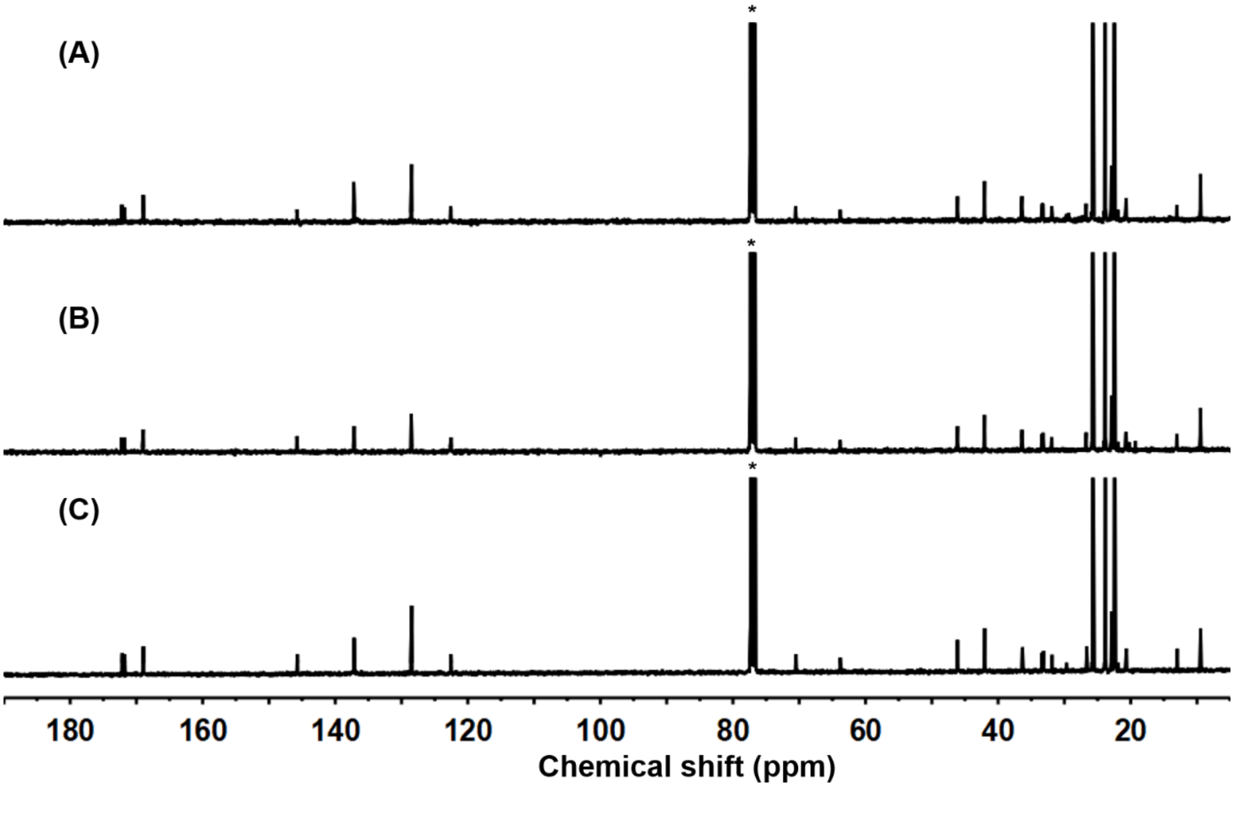

Figure S2. ^{13}C NMR spectra of (A) *para*-B_2VB_2, (B) *meta*-B_2VB_2, and (C) *ortho*-B_2VB_2. Asterisks at 77 ppm are resonances from residual $CHCl_3$.

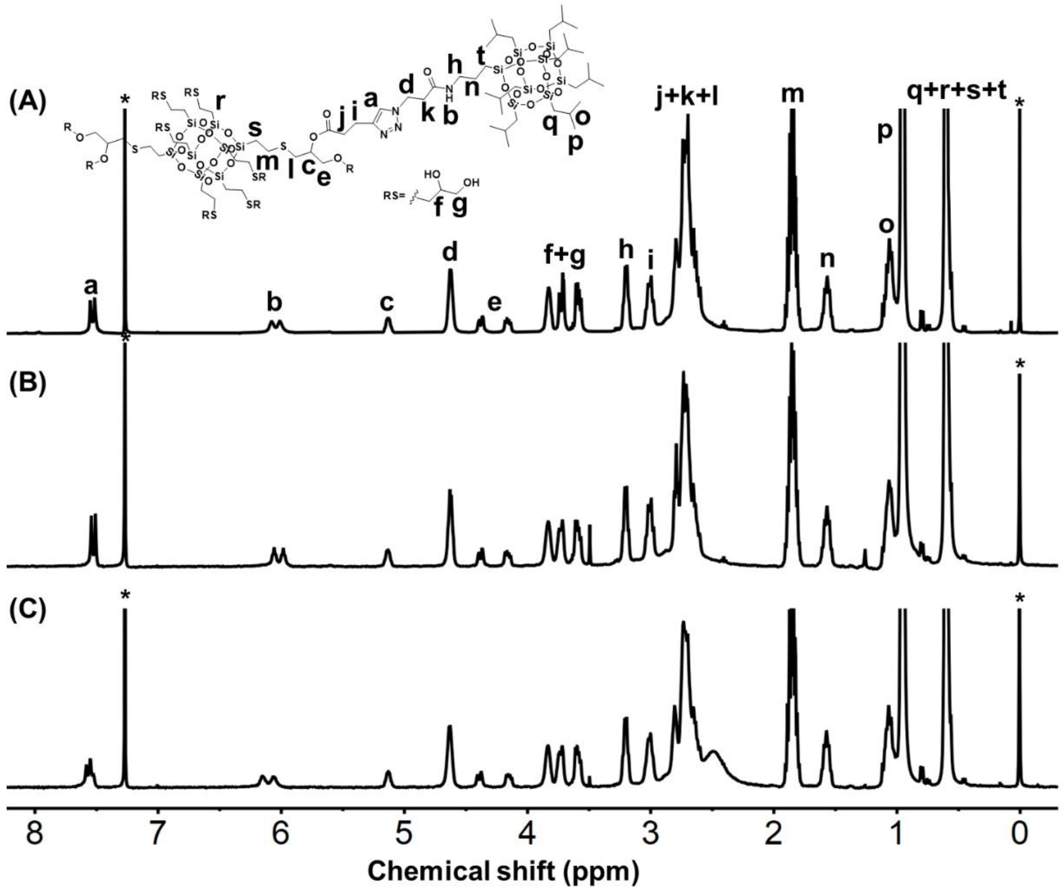

Figure S3. ^{1}H NMR spectra of (A) *para*-B_2DB_2, (B) *meta*-B_2DB_2, and (C) *ortho*-B_2DB_2. Asterisks at 7.27 ppm and 0 ppm are resonances from residual $CHCl_3$ and TMS.

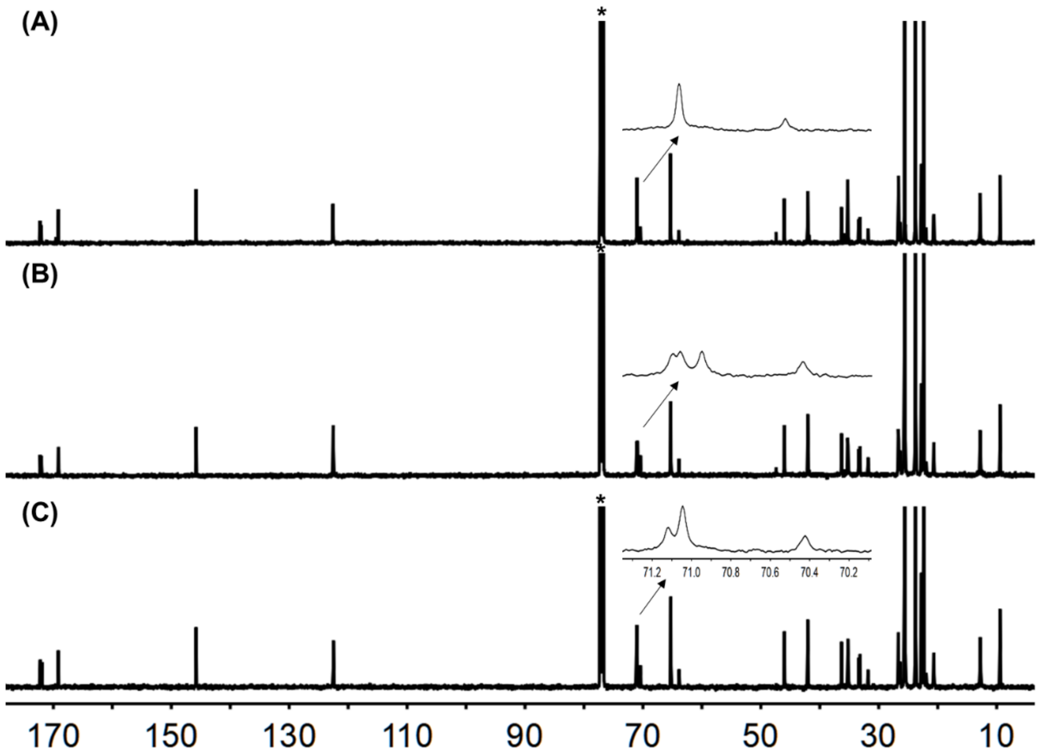

Figure S4. ^{13}C NMR spectra of (A) *para*-B_2DB_2, (B) *meta*-B_2DB_2, and (C) *ortho*-B_2DB_2. Asterisks at 77 ppm are resonances from residual $CHCl_3$.

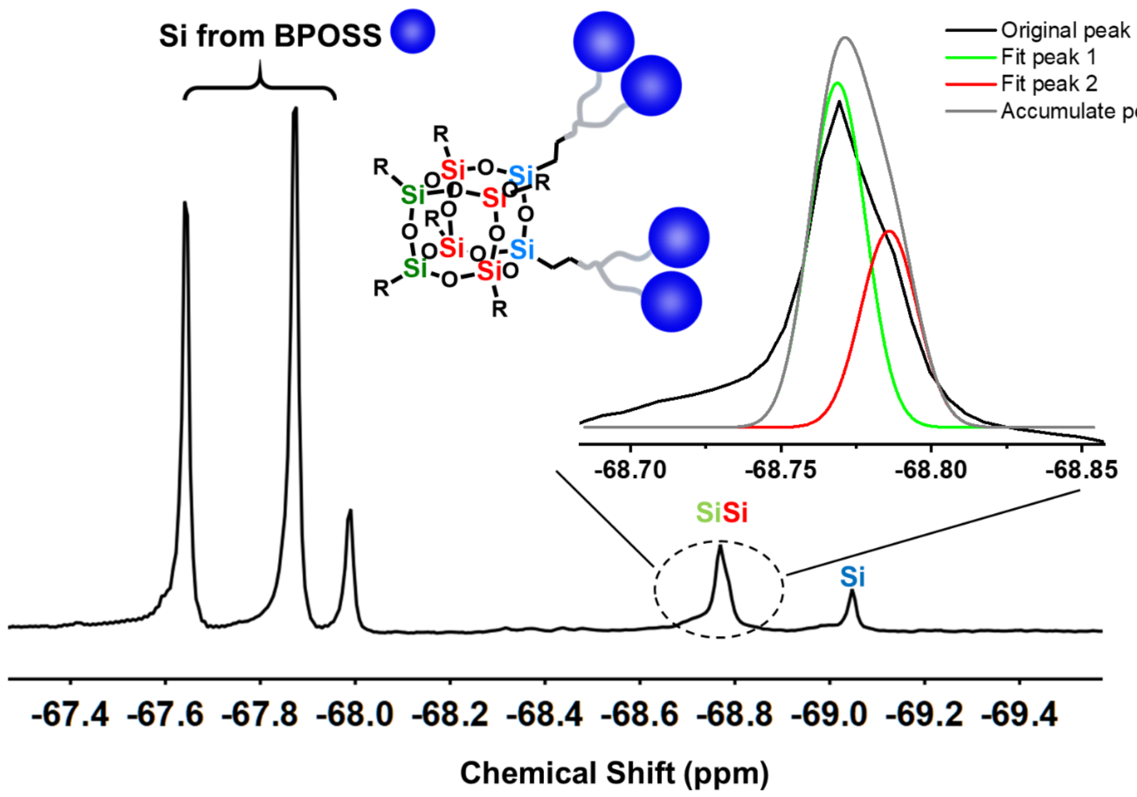

Figure S5. ^{29}Si NMR spectrum of *ortho*-B_2DB_2 and the peak analysis, the integral area of peak 1 and 2 is 1.74:1 which is approximately 2 to 1.

Section II. Molecular Self-assemblies.

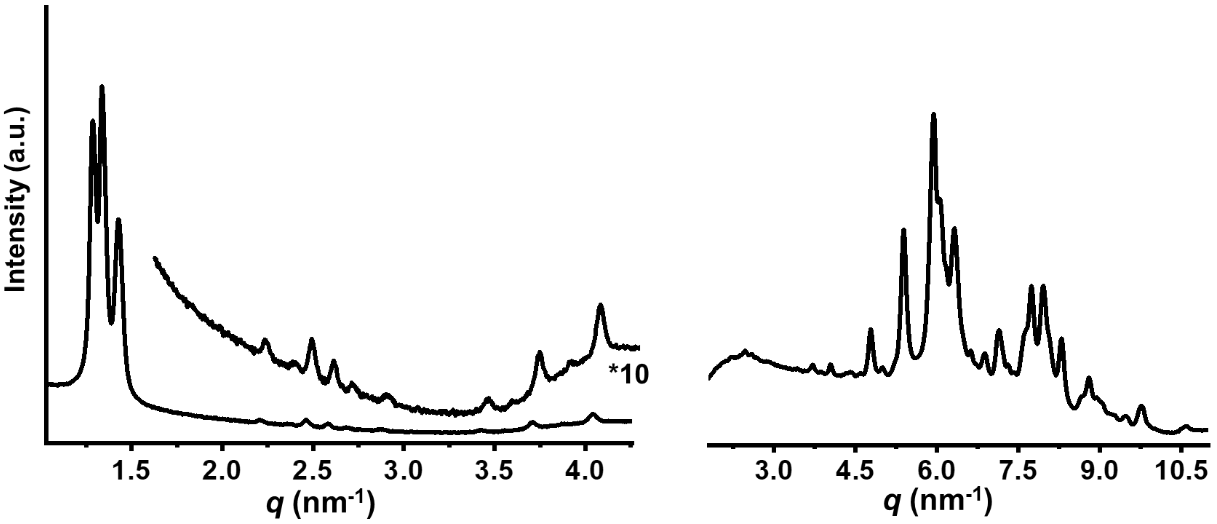

Figure S6. Synchrotron X-ray scattering patterns of *para*-B_2DB_2 at the small (left) and wide (right) angle regions.

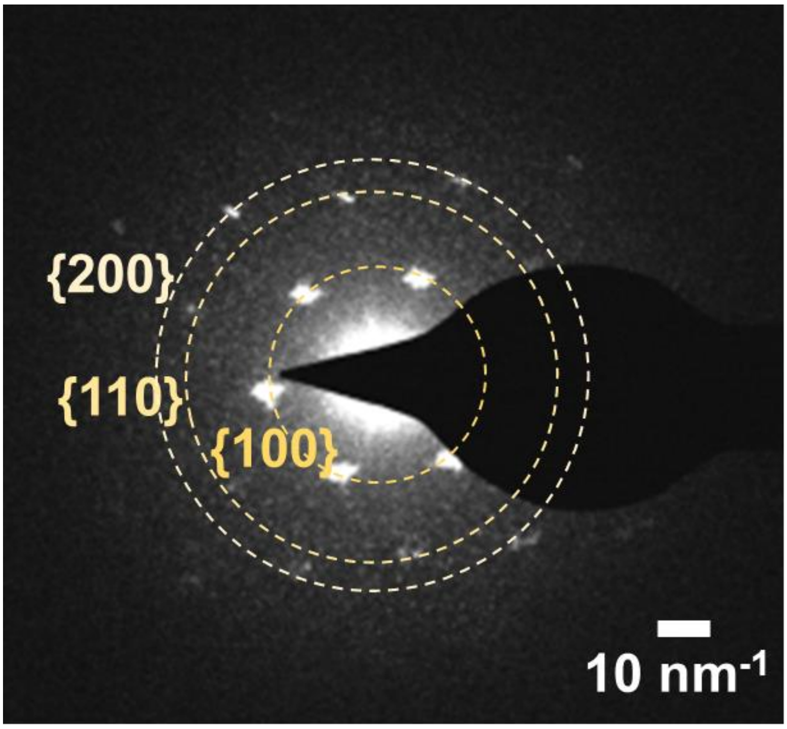

Figure S7. Selected area electron diffraction (SAED) pattern of *para*-B_2DB_2 along <001> direction of hexagonal lattice from Figure 3D.

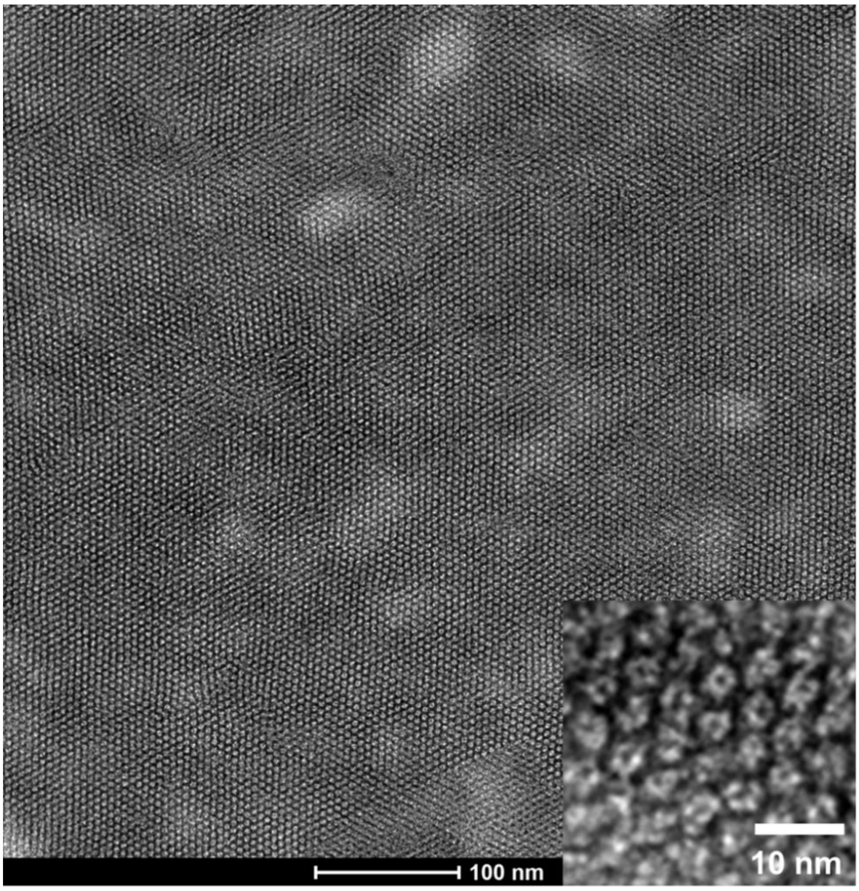

Figure S8. Dark field TEM image of *para*-B_2DB_2 annealed at 175 °C using the same sample imaged in Figure 3D.

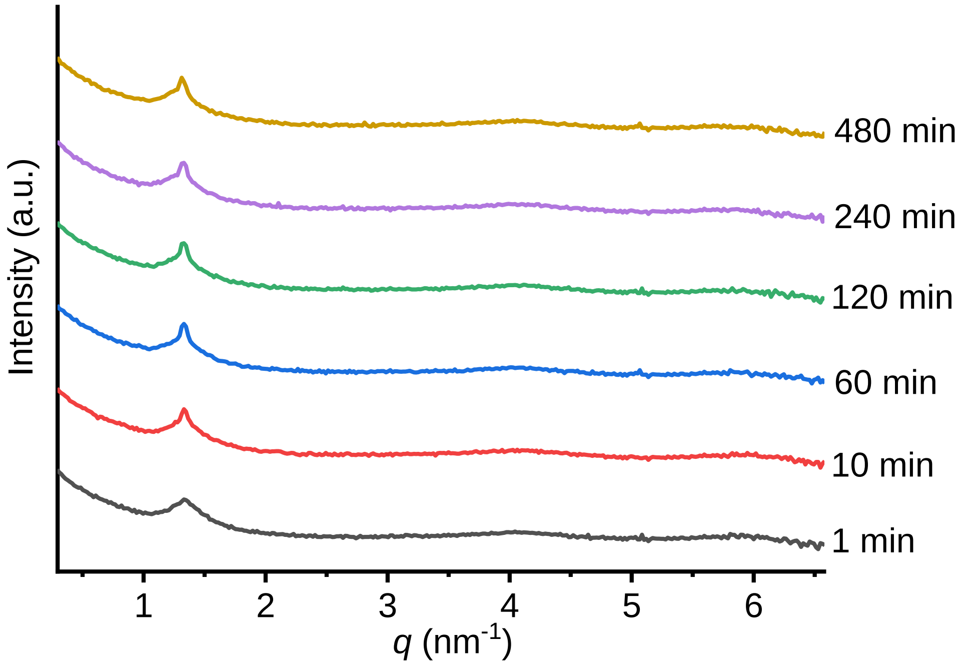

Figure S9. SAXS pattern of *meta*-isomer after melted at 200 °C for 1 min and annealed at 180 °C over a period of time. Only one broad peak with a sharp roof was detected at ~1.3 nm^{-1} which means that a metastable phase was formed.

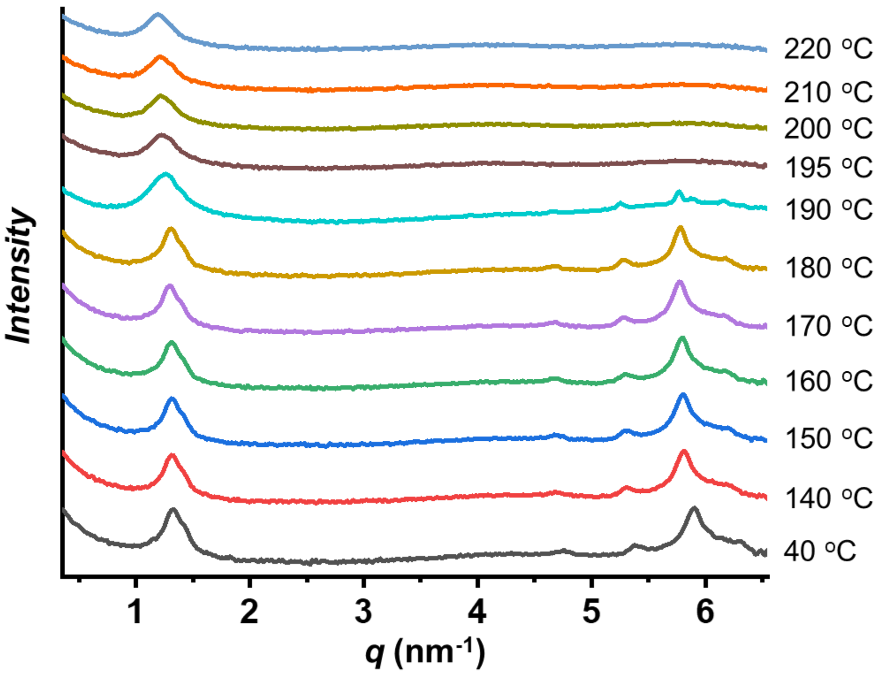

Figure S10. SAXS pattern of *ortho*-B_2DB_2 heated up from 40 °C to 220 °C (10 °C/min) after shearing at 188 °C. The hexagonal crystal is formed after shearing, however, it can hardly form the A15 phase upon the melting of BPOSS. The temperature is hold for at least 30 min per interval.

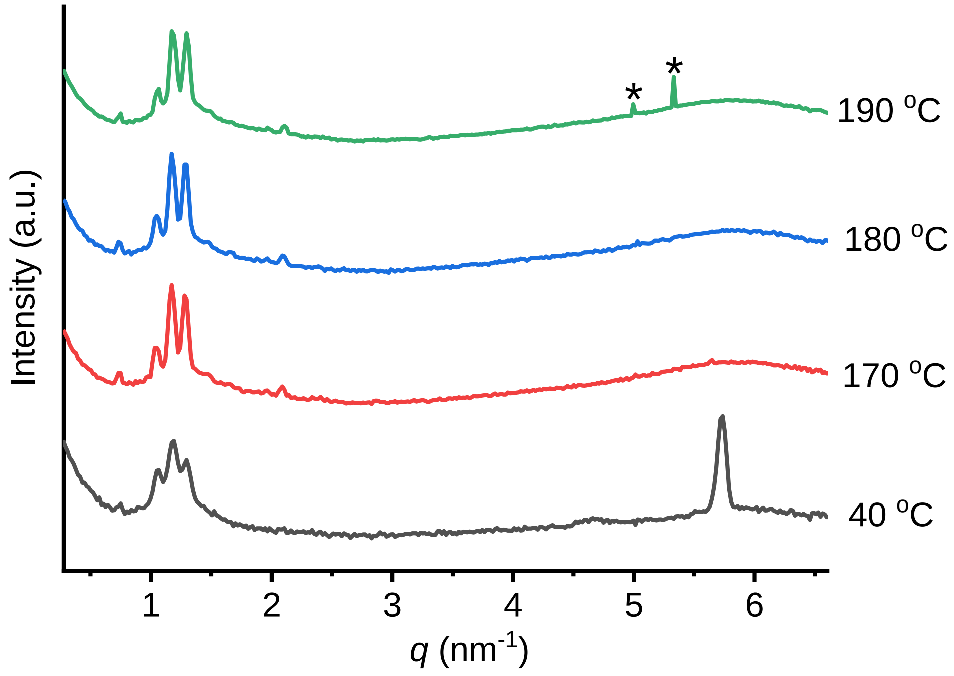

Figure S11. SAXS pattern of *ortho*-B_2DB_2 after forming the A15 phase at 190 °C then cooling down to 40 °C (10 °C/min), the A15 phase will not translate to hexagonal crystal while the crystallinity of BPOSS is also very low with only one peak at ~ 5.7 nm^{-1}. The temperature was hold for at least 30 min per interval. Asterisks at ~ 5.5 nm^{-1} are noisy points of some bad pixels from the detector.

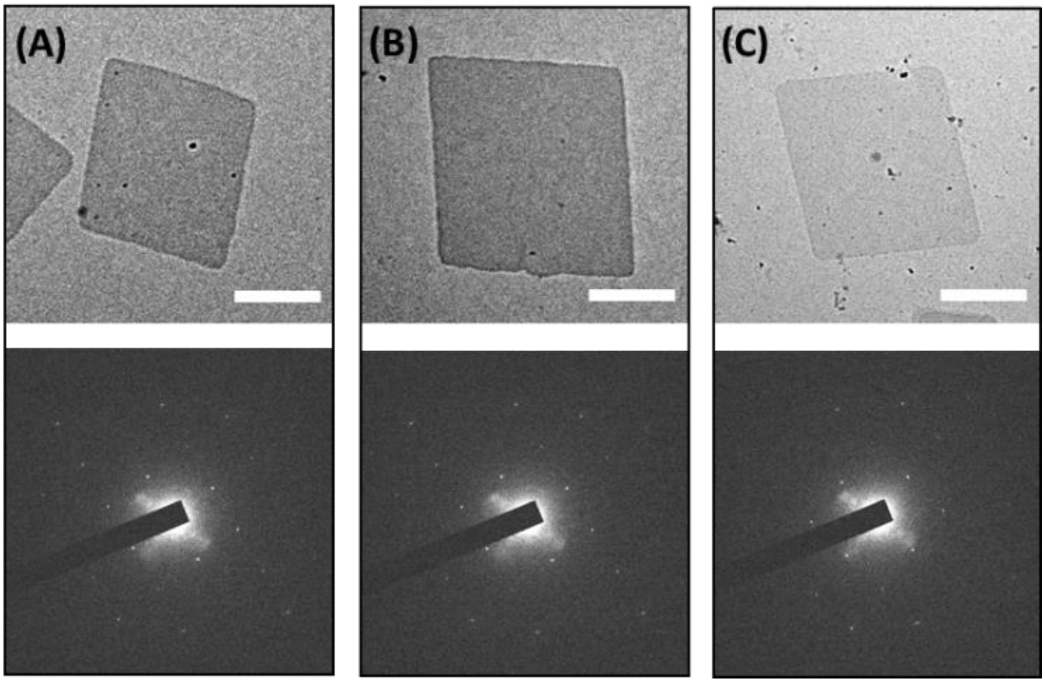

Figure S12. TEM images of the 2D nanosheets grown from highly diluted solution for *para*-B_2DB_2 (A), *meta*-B_2DB_2 (B), and *ortho*-B_2DB_2 (C), and the corresponding SAED patterns (bottom ones). The scale bar is 1 µm.

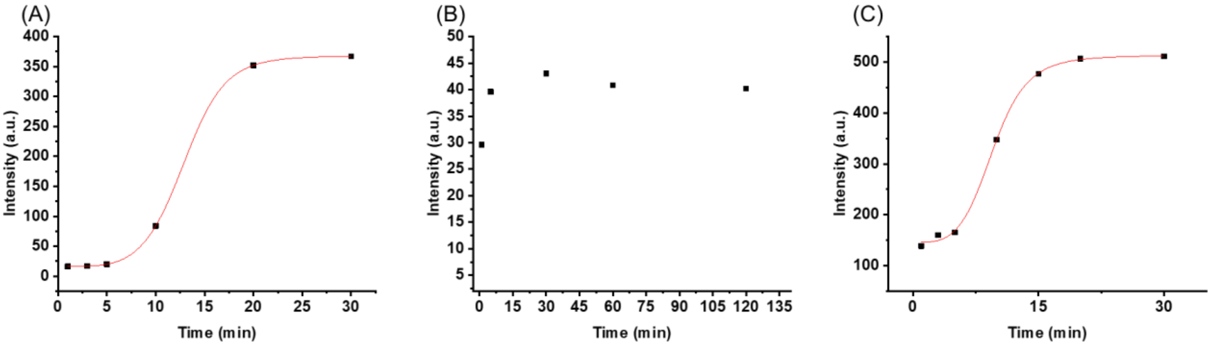

Figure S13. Correlation of first-order peak intensity and annealing time after quenching from isotropic to 180 °C for *para*-isomer (A, $t_{1/2}$=14.6 min from the red fitting curve) and *meta*-isomer (B), and correlation of 210 peak intensity and annealing time after quenching from isotropic to 185 °C for *ortho*-isomer (C, $t_{1/2}$=10.7 min from the red fitting curve) showing the self-assembly kinetics.

Section III. Calculations.

Volume fraction (f):

$$f_{\text{DPOSS}} = \frac{(M_{n(\text{DPOSS})}/\rho_{\text{DPOSS}})}{(M_{n(\text{DPOSS})}/\rho_{\text{DPOSS}})+M_{n(\text{BPOSS})}/\rho_{\text{BPOSS}})+(M_{n(\text{Linker})}/\rho_{\text{Linker}})} \quad \text{Eq. S1}$$

$$f_{\text{BPOSS}} = \frac{(M_{n(\text{BPOSS})}/\rho_{\text{BPOSS}})}{(M_{n(\text{DPOSS})}/\rho_{\text{DPOSS}})+M_{n(\text{BPOSS})}/\rho_{\text{BPOSS}})+(M_{n(\text{Linker})}/\rho_{\text{Linker}})} \quad \text{Eq. S2}$$

$$f_{\text{Linker}} = 1 - f_{\text{DPOSS}} - f_{\text{BPOSS}} \quad \text{Eq. S3}$$

where the molecular weight of the BPOSS building block $M_{\text{n,BPOSS}}$ is 816.5 g/mol which should be times four during the calculation, the molecular weight of the DPOSS building block $M_{\text{n,DPOSS}}$ is 1227.9 g/mol, the molecular weight of the linker $M_{\text{n,Linker}}$ is 605.7 g/mol which should be times two during the calculation. The density values are 1.13 g/cm^3 for BPOSS, 1.43 g/cm^3 for DPOSS and 1.16 g/cm^3 for linker.[3] Then, the calculated f_{DPOSS} is 0.18, the calculated f_{BPOSS} is 0.60, the calculated f_{Linker} is 0.22 which is added to BPOSS part when did further calculations.

Number of molecules in specific space (μ):

For Col$_h$,

$$\mu = \frac{\pi(\frac{d}{2})^2 r\rho}{M} \times N_{\text{A}} \quad \text{Eq. S4}$$

where d is the columnar diameter in Col$_h$ lattices, r is the selected space (1 nm), ρ is the density of the sample (1.24g/cm^3 for *para*-isomer and 1.22g/cm^3 for *meta*-isomer), M is molecular weight of sample and N_{A} is the Avogadro's number.

For A15 phase,

$$\mu = \frac{a^3\rho}{8M} \times N_A \quad \text{Eq. S5}$$

where lattice dimension a can be calculated as $d_{110} \times \sqrt{2}$ which is 11.8 nm, molecular weight (M) of B_2DB_2 is 5705 g/mol, ρ is 1.23 g/cm^3, N_A is Avogadro constant, 6.02×10^{23}.

Average cross-section area per molecule (A_0):

For Col$_h$, the cross-section area (A_0) can be calculated as:

$$A_0 = \frac{4M_{\mathrm{BPOSS}}\sqrt{1-f_{\mathrm{BPOSS}}}}{Rf_{\mathrm{BPOSS}}\rho_{\mathrm{BPOSS}}N_{\mathrm{A}}} \qquad \text{Eq. S6}$$

where R is the intercolumn distance of Col$_h$ ($R = \frac{2d_1}{\sqrt{3}}$).

For A15,

$$A_0 = \frac{4\pi(\frac{D}{2})^2}{\mu} \qquad \text{Eq. S7}$$

$$D = \sqrt[3]{\frac{3f_{\mathrm{DPOSS}}}{4\pi}}a \qquad \text{Eq. S8}$$

where D is the average diameter of DPOSS aggregates in each sphere of the A15 lattice and a is the lattice parameter of A15.

Full indexation and lattice parameters of hexagonal phase may be determined by solving Eq. S9:

$$\frac{1}{d_{hkl}^2} = \frac{4}{3}\left(\frac{h^2+k^2+l^2}{a^2}\right) + \frac{l^2}{c^2} \qquad \text{Eq. S9}$$

the indexation is listed in Table S1-3.

Full indexation and lattice parameters of cubic A15 phase may be determined by solving Eq. S10:

$$\frac{1}{d_{hkl}^2} = \frac{h^2+k^2+l^2}{a^2} \qquad \text{Eq. S10}$$

The indexation is listed in Table S4.

Table S1. SAXS peak assignments for *para*-B_2DB_2 superlattice.

Hexagonal, *a*=5.76 nm, *c*=9.59 nm<br>*c*/*a* = 1.665 (ideal: 1.633)		
(*hkl*)	q_{obs} †	q_{calc} ‡
100	1.26	1.26
002	1.31	1.31
101	1.41	1.42
110	2.20	2.19
103	2.35	2.34
200	2.46	2.52
201	2.58	2.61
004	2.68	2.62
202	2.87	2.84
211	3.43	3.40
212	3.55	3.59
300	3.71	3.79
301	3.87	3.84
302	4.04	4.01

† Peak positions from SAXS data. ‡ Expected peak positions for assigned space group with given lattice dimensions.

Table S2. SAXS peak assignments for *meta*-B_2DB_2 superlattice.

Hexagonal, *a*=5.76 nm, *c*=9.59 nm<br>*c*/*a* = 1.665 (ideal: 1.633)		
(*hkl*)	q_{obs} †	q_{calc} ‡
100	1.26	1.26
002	1.31	1.31
101	1.41	1.42
103	2.38	2.34
212	3.56	3.59

† Peak positions from SAXS data. ‡ Expected peak positions for assigned space group with given lattice dimensions.

Table S3. SAXS peak assignments for *ortho*-B_2DB_2 superlattice.

Hexagonal, a=5.58 nm, c=9.38 nm<br>c/a = 1.681(ideal: 1.633)		
(hkl)	q_{obs}†	q_{calc}‡
100	1.30	1.30
002	1.34	1.34
101	1.45	1.46

† Peak positions from SAXS data. ‡ Expected peak positions for assigned space group with given lattice dimensions.

Table S4. SAXS peak assignments for *ortho*-B_2DB_2 with A15 phase.

Cubic, ($Pm\bar{3}n$), a=11.07 nm		
(hkl)	q_{obs}†	q_{calc}‡
110	0.803	0.803
200	1.14	1.14
210	1.27	1.27
211	1.39	1.39
220	1.60	1.61
310	1.79	1.79
320	2.04	2.05
321	2.12	2.12
400	2.27	2.27
410	2.34	2.34

† Peak positions from SAXS data. ‡ Expected peak positions for assigned space group with given lattice dimensions.

Table S5. Key parameters that influence the self-assembly behavior and phase structure of isomeric giant molecules.[1,2,4]

BDB[a]	f_B[c]	phase	A_0 nm[2] [c] (Temp.°C)	B_2DB_2[a]	f_B	phase	A_0 nm[2] (Temp. °C)
p	0.75	LAM	2.37(170)	*p*	0.82	HEX	2.21(186)
m		-	-	*m*		-	-
o		DG	2.05(160)	*o*		A15	2.16(186)
SDS[b]	f_S	phase	A_0 nm[2] (Temp. °C)	SDS[b]	f_S	phase	A_0 nm[2] (Temp. °C)
p	*N*=11 0.73	LAM	2.06(120)	*p*	*N*=35 0.88	BCC	1.94(120)
m		DG	1.79(120)	*m*		BCC	1.94(120)
o		DG	1.78(120)	*o*		BCC	1.92(120)
SDS[b]	f_S	phase	A_0 nm[2] (Temp. °C)	S_2DS_2[b]	f_S[c]	phase	A_0 nm[2] [c] (Temp. °C)
p	*N*=20 0.82	HEX	1.95(120)	*p*	*N*=11 0.87	HEX	2.35(100)
m		HEX	1.89(120)	*m*		HEX	2.13(100)
o		HEX	1.88(120)	*o*		σ	2.03(120)

[a] B stands for hydrophobic BPOSS, D stands for hydrophilic DPOSS. [b] S stands for hydrophobic PS. [c] These volume fraction values and the corresponding A_0 values were recalculated to be consistent for the comparison. In all the calculations, linkers were assigned to the hydrophobic part such as B or S. *N* is the degree of polymerization of the tethered polystyrene chains.

References

1. Han, D.; Shao, Y.; Tao, Y.-D.; Han, G.; Zhou, D.-L.; Yang, S.; Zhang, W.-B.; Fu, Q., Symmetry-guided, divergent assembly of regio-isomeric molecular Janus particles. *Chem. Commun.* **2019,** *55*, 6425-6428.
2. Shao, Y.; Han, D.; Yan, X.; Hou, B.; Li, Y.; He, J.; Fu, Q.; Zhang, W.-B., Phase Behaviors of Multi-tailed B_2AB_2-Type Regio-isomeric Giant Surfactants at the Columnar-Spherical Boundary. *Chin. J. Chem.* **2021,** *39*, 3261-3268.
3. Feng, X.; Zhang, R.; Li, Y.; Hong, Y. L.; Guo, D.; Lang, K.; Wu, K. Y.; Huang, M.; Mao, J.; Wesdemiotis, C.; Nishiyama, Y.; Zhang, W.; Zhang, W.; Miyoshi, T.; Li, T.; Cheng, S. Z. D., Hierarchical Self-Organization of AB_n Dendron-like Molecules into a Supramolecular Lattice Sequence. *ACS Central Sci.* **2017,** *3*, 860-867.
4. Wang, X.-M.; Shao, Y.; Jin, P.-F.; Jiang, W.; Hu, W.; Yang, S.; Li, W.; He, J.; Ni, P.; Zhang, W.-B., Influence of Regio-Configuration on the Phase Diagrams of Double-Chain Giant Surfactants. Macromolecules **2018**, *51*, 1110-1119.
